# Trends in in ovo sexing technologies: insights and interpretation from papers and patents

**DOI:** 10.1186/s40104-023-00898-1

**Published:** 2023-07-14

**Authors:** Matthias Corion, Simão Santos, Bart De Ketelaere, Dragana Spasic, Maarten Hertog, Jeroen Lammertyn

**Affiliations:** 1 KU Leuven, BIOSYST-MeBioS Biosensors Group, Willem de Croylaan 42, Leuven, B-3001 Belgium; 2 KU Leuven, BIOSYST-MeBioS Biostatistics Group, Kasteelpark Arenberg 30, Leuven, B-3001 Belgium; 3 KU Leuven, BIOSYST-MeBioS Postharvest Group, Willem de Croylaan 42, Leuven, B-3001 Belgium

**Keywords:** Animal welfare, In ovo sexing, Male day-old chick culling, Optical and non-optical techniques, Patents and papers

## Abstract

Numerous researchers and institutions have been developing in ovo sexing technologies to improve animal welfare by identifying male embryos in an early embryonic stage and disposing of them before pain perception. This review gives a complete overview of the technological approaches reported in papers and patents by performing a thorough search using Web of Science and Patstat/Espacenet databases for papers and patents, respectively. Based on a total of 49 papers and 115 patent families reported until May 2023 worldwide, 11 technology categories were defined: 6 non-optical and 5 optical techniques. Every category was described for its characteristics while assessing its potential for application. Next, the dynamics of the publications of in ovo sexing techniques in both paper and patent fields were described through growth curves, and the interest or actual status was visualized using the number of paper citations and the actual legal status of the patents. When comparing the reported technologies in papers to those in patents, scientific gaps were observed, as some of the patented technologies were not reported in the scientific literature, e.g., ion mobility and mass spectrometry approaches. Generally, more diverse approaches in all categories were found in patents, although they do require more scientific evidence through papers or industrial adoption to prove their robustness. Moreover, although there is a recent trend for non-invasive techniques, invasive methods like analyzing DNA through PCR or hormones through immunosensing are still being reported (and might continue to be) in papers and patents. It was also observed that none of the technologies complies with all the industry requirements, although 5 companies already entered the market. On the one hand, more research and harmony between consumers, industry, and governments is necessary. On the other hand, close monitoring of the market performance of the currently available techniques will offer valuable insights into the potential and expectations of in ovo sexing techniques in the poultry industry.

**Supplementary Information:**

The online version contains supplementary material available at 10.1186/s40104-023-00898-1.

## Introduction

Currently, much attention is given to producing safe and clean food. Although there has been a change in diets in developed countries, causing a decrease in meat consumption per capita, global meat consumption is still significantly increasing due to the development of underdeveloped countries and population increase [1]. This increased demand for food, including chicken eggs and meat, has led to a high specialization of chicken breeds (i.e., genetic lines providing high food quantities with the least resources possible) and extensive poultry industry mechanization [2]. However, male chicks have no use in the industry since they do not lay eggs, and their meat is not appreciated by typical consumers [3]. Hence, male chicks are killed upon hatching (known as male chick culling) and used for other purposes, e.g., zoo animal feed [1]. Nevertheless, male culling raises ethical and economic issues among consumers, who prefer food from animal-friendly methods [4], and among the industry which sees the culling as an economic burden since half of the incubators (culled males) hardly generates any revenue [1, 2]. Therefore, in some European countries (e.g., Germany [5], France [6], and Italy [7]), there is an agenda for a more environmentally and animal-friendly poultry industry. In Germany, culling has been prohibited since the beginning of 2022 [5].

In this context, 2 solutions were proposed to tackle the chick culling issue: 1) raising males from layer breeds or dual-purpose chickens with males and females being used for respectively meat and eggs, although this results in a higher environmental impact and mainly serves niche market purposes since most consumers do not appreciate the product [8]; and 2) in ovo sexing – methods used to determine the embryo’s sex before hatching. Even though the technology cost can influence the final price for consumption eggs, surveys showed that this latter solution pleases the consumers (when performed in an early incubation stage, i.e., before the onset of embryonic pain perception) and the industry the most, allowing them to use male eggs for animal feed and to fill the incubators with only females [4]. Regarding pain perception, it is accepted that this might start after d 7 when the multisynaptic reflex arches are closed, making the embryo sensitive to mechanical stimuli [9]. However, encephalogram signals are visible only after d 12, and it is expected that chicken embryos will start feeling pain from that moment onwards [10]. Table 1 depicts the complexity in the laying hen industry by summarizing the advantages and disadvantages of the current situation (i.e., culling of day-old male chicks) versus in ovo sexing (above explained as the solution with the most promising future) [1].

**Table 1:** Advantages and disadvantages of the current situation in the laying hen industry versus in ovo sexing

	**Current situation**		**In ovo sexing**	
	**Advantages**	**Disadvantages**	**Advantages**	**Disadvantages**
**Consumer**	Low prices	Unethical	Ethical	Higher prices
**Industry**	Selling corpses as feed	Gruesome working conditions No profit on male chicks	Ethical Re-purposing culled eggs Incubator space optimization	High investments
**Male chick*s***	NA	Naturalness infringement^a^ Pain and stress	No/less pain or stress	Possible pain perception depending on incubation day
**Environment**	Efficient production No waste	More energy necessary for incubation	Lower electricity consumption	Consumables waste

Information adapted from Bruijnis et al. [1]

*NA* Non-applicable

^a^Naturalness is one of the Five Freedoms in animal welfare, in which an animal should be allowed to demonstrate their animal behavior [11]. With culling male chicks, this possibility is not given to the male chicks. The culling is closely related to the lack of animal integrity the sector gives these male chicks, rendering as a non-ethical practice

Nevertheless, to gain broad market acceptance, in ovo sexing technologies must possess the following characteristics: 1) work with all colors of eggs (e.g., white and brown eggs), 2) present accuracies higher than 98.5%, (i.e., the same accuracy of a sexing expert), 3) identify the sex at the earliest possible stage (preferably before possible onset of pain), 4) have high throughput (20,000 to 30,000 eggs/h), 5) avoid disturbing the embryos or their hatchability, and 6) have a low impact on the production cost [1, 12]. Currently, only a few technologies are being used in the industry, even though they do not comply with all the mentioned requirements.

**Fig. 1:**
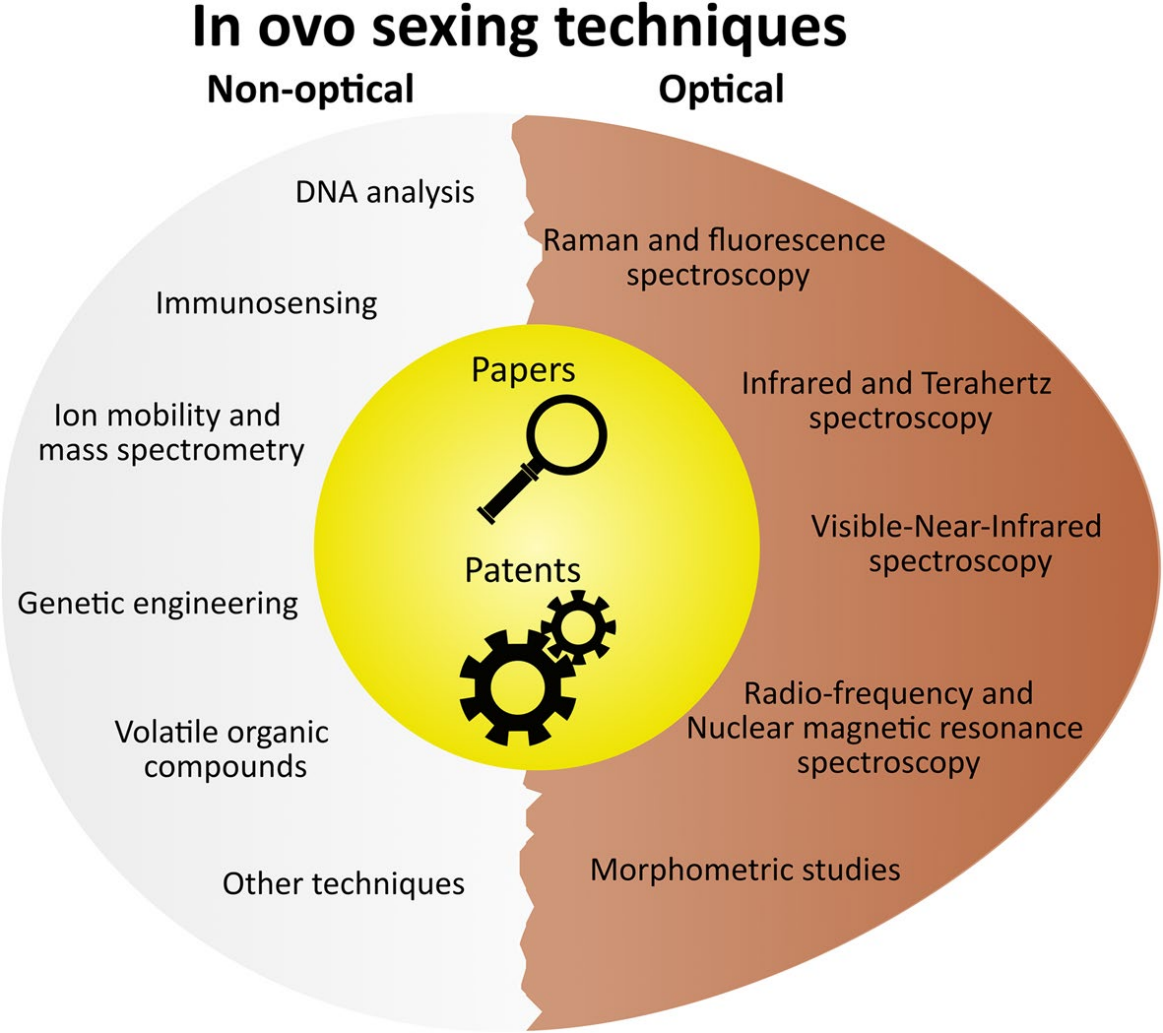
Representation of the review structure. The papers and patents were divided into 6 non-optical and 5 optical methods of in ovo sexing

This review intends to give an overview of the history and current trends of in ovo sex identification techniques. Moreover, unlike previous reviews, this work combines the paper and patent literature and aims to reveal the in-depth relationship between innovations from the academic and industrial side. A conventional qualitative analysis based on scientific content is followed by an overview of the commercially applied in ovo sexing techniques nowadays, and a quantitative analysis of the number of papers and patents together with their respective number of citations and legal status. The search method and final search keys can be found in Section S1 of the Supplementary materials. Similar to the latest review on in ovo sexing techniques by Krautwald-Junghanns et al. [13], we grouped our 11 technology categories into 6 non-optical and 5 optical techniques (Fig. 1).

## Non-optical in ovo sexing methods

The non-optical in ovo sexing technologies (summarized in Table 2) mainly comprise invasive approaches, such as sample extraction, marker insertion, or shell windowing for vessel screening. This section includes a discussion on 1) DNA analysis and 2) immunosensing, both being standard in ovo sexing methods, 3) ion mobility spectrometry (IMS) and mass spectrometry (MS; measuring the presence of hormones or metabolites), an overview of 4) genetic engineering or genetically modified organisms (GMO) and 5) Volatile Organic Compounds (VOC) as gases emitted through the eggshell, next to a compilation of 6) other techniques not included in the previous 5 categories.

**Table 2:** Overview of non-optical in ovo sexing techniques

**Category**	**Target**	**Incubation day/ Accuracy/ Throughput**	**Technology (patents)/ Research gaps (papers)**	**Advantages (+)/ Drawbacks (-)**	**References**
DNA analysis	gDNA (e.g., *CHD*, *Xho*I, *SWIM*, *HINTW*) from different samples (e.g., AF, blood, feathers, brain)	d 8 to 13/ 100%/ Slow (slower in case of need for DNA extraction), PCR can take more than 60 min	Technology > Research Lack of papers using AF Several possible female-specific sequences are not reported in the literature	** + ** High accuracy Early in incubation (> d 2.5) **-** Lengthy processes and expensive Can be highly invasive if not using AF	**Patents** □ Griffiths [14], □ Clinton [15], ○ Dalton et al. [16], ○ Arie and Danielli [17], ◊ Molina and Espeut [18], ○ Weigel et al. [19] **Papers** Li et al. [20], Haunshi et al. [21], Clinton et al. [22], He et al. [23], Petitte and Kegelmever [24]
Immunosensing	Sexual hormones (e.g., E2 or estrone-3-sulfate) in AF or blood	Minimum d 8/ Reported as 99% on d 9/ Relatively slow using ELISA/RIA, which can take up to 90 min	Technology ≅ Research Well defined in both papers and patents	** + ** Applicable in AF **-** Limited to after d 8 of incubation Lower accuracies than DNA analysis Expensive and lengthy testing	**Patents** ◊ Tyczkowski et al. [25], ○ Butt and Tran [26], ○ Einspanier [27, 28], □ Phelps [29], ◊ Meter [30, 31] **Papers** Phelps et al. [12], Tanabe et al. [32], Weissmann et al. [33, 34], Müller et al. [35], Aslam et al. [36], Wang et al. [37]
IMS and MS	Metabolites in AF	d 9 to 10/ 90% to higher than 95%/ Relatively fast	Technology > Research Only defined by 2 different groups of inventors in the patent literature No work has been published in the scientific literature	** + ** Quick sampling and relatively fast analysis **-** No scientific literature on physiological background	**Patents** □ Daum and Atkinson [38], ○ Bruins and Stutterheim [39, 40], ◊ Stutterheim et al. [41–43] **Papers** -
Genetic engineering	gDNA	d 0 to 2.5/ 100%/ High if using labels	Technology > Research Only one short communication in papers, mainly present in patents	** + ** High accuracies at an early incubation stage **-** Not accepted in several countries (see text) Invasive injection of a labeled molecule (antibody/DNA sequence)	**Patents** ◊ Decuypere and Fey [44], ○ Offen [45], □ Beisswanger [46] **Papers** Doran et al. [47], Lee et al. [48]
VOC analysis	VOCs released from the egg	d 0 or during incubation/ Accuracy not defined/ s to min	Technology > Research Patents use real-time analysis systems such as THz spectroscopy, SIFT- or PTR-MS Research papers use conventional SPME–GC–MS analysis and more research is required for generating robust sexing models	** + ** Non-invasive Potential for early-stage application **-** Contamination risk of egg odor by environment or other eggs	**Patents** ◊ Rivers [49], ○ Gabbai [50], ○ Knepper et al. [51], ○ Shi et al. [52] **Papers** Webster et al. [53], Costanzo et al. [54], Xiang et al. [55]
Other	1) Electrical and magnetic polarity 2) Dynamic electromagnetic spectrum 3) Sex-specific compounds close to the eggshell 4) Bioimpedance measurements	d 0 or d 9/ Accuracies unknown/ No throughputs defined	Technology > Research Only defined in different patents and no scientific literature	** + ** Before incubation Non-invasive **-** Little scientific evidence	**Patents** □ Young [56], □ Williams [57, 58], □ Chadfield [59], □ Pacala et al. [60], ○ Visser [61], ◊ Rastoutsau et al. [62] **Papers** Ching et al. [63]

*d* Day, *gDNA* Genomic DNA, *AF* Allantoic fluid, *E2* Estradiol, *ELISA* Enzyme-linked immunosorbent assay, *RIA* Radioimmunoassay, *THz* Terahertz, *SPME–GC–MS* Solid phase micro extraction–gas chromatography-mass spectrometry, *SIFT-MS* Selected ion flow tube-mass spectrometry, *PTR-MS* Proton transfer reaction-mass spectrometry, *IMS and MS* Ion mobility and mass spectrometry, ○ Granted, ◊ Pending, □ Ceased/Rejected/Discontinued

### DNA analysis

**Fig. 2:**
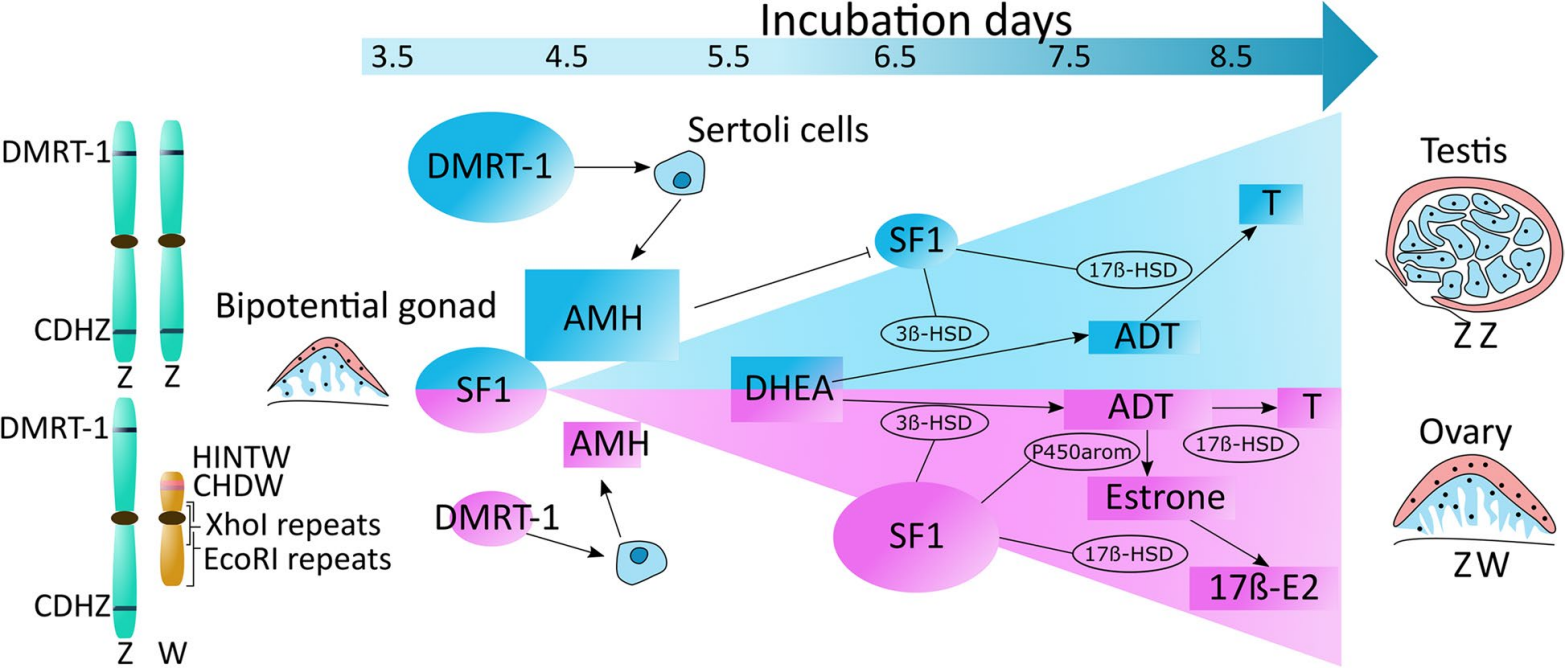
Representation of the ZZ and ZW genes in males and females and gonadal development. The Anti-Mullerian hormone has a significant role in males by inhibiting the *SF1* gene. In females, the *SF1* gene is less inhibited, forming several proteins (3β-HSD, P450arom and 17β-HSD), fundamental for the estrogens-related products (e.g., 17β-E2 or estrone-3-sulfate). Until d 3.5 of incubation, there is no sexual differentiation, whereas gene expression and hormonal presence differences are noticed after this moment [68, 69]. *SF1 * Steroidogenic factor 1, *AMH* Anti-Mullerian hormone, *DHEA* Dehydroepiandrosterone, *ADT* Androstenedione, *HSD* Hydroxysteroid dehydrogenases

DNA analysis using polymerase chain reaction (PCR) followed by band observation with gel electrophoresis has been used as an in ovo sexing technique relying on the sexual gametes heterogeneity between males (ZZ) and females (ZW) [64]. Due to this heterogeneity, it is possible to perform sexual detection using W chromosome genes. In the literature, 7 distinct genes have been reported (Fig. 2): doublesex and mab-3 related transcription factor 1 (*DMRT-1*) [23] and chromodomain helicase DNA (*CHD*)*-Z* from the Z-chromosome, and *CHD-W* [20], W-protein kinase C iota (*WPKCI)* [65], *Xho*I family [24] and *SWIM* [23] from the W-chromosome, and the expression of the *JUN* gene [66, 67] located on chromosome 8. Interestingly, this detection method has been applied using distinct types of tissues from the embryo (e.g., blood, muscle, feathers, or brain).

Griffiths [14] patented the first female-specific gene for in ovo sex identification (discontinued in 2004), using *CHD* from genomic DNA (gDNA) (the patent also included antibodies use for the encoded proteins, which lays outside of this section’s scope). A paper by Li et al. [20] aligns with the claims in the previous patent using the sequences *CHDZ* and *CHDW* with several duck species’ samples such as blood and feathers (adults), chorioallantoic membrane (d 13), and allantoic fluid (AF) (d 8, 10 and 13). The report’s main outcome was an assay with a 100% correct identification rate based on 256 female and 256 male analyzed samples.

Another example of a commonly used gene is the *Xho*I repetition family. Petitte and Kegelmever [24] published one of the first papers on this, showing that *Xho*I was specific for female samples, enabling DNA detection as low as 2 cells per sample. Further, other *Xho*I repetition family examples were presented by Haunshi et al. [21] and Clinton et al. [22], the latter using an isothermal PCR-free approach and fluorescence resonance energy transfer (FRET) method in blood samples, with real-time results observation in 15 min.

Another possibility was presented when Clinton [15] protected a new method claiming a new nucleic acid probe to detect *WPKCI* in AF or amniotic fluid, tissue, and organs. Furthermore, in He et al. [23], quantitative PCR (qPCR) and a new female-specific gene sequence (i.e., *SWIM*) were used for the first time for in ovo sexing on d 9. The results showed no misidentification and a low detection limit (as low as 1 ng of total DNA) with crude brain samples.

In conclusion, so far, the methods in papers rely on highly invasive samples (i.e., inadequate for in ovo sexing). Contrary to this, AF is an extra-embryonic sample offering relatively easy sampling, low invasiveness and low hatchability effects. Furthermore, the industry has higher demand for non-invasive methods, compared to research. Thus, except for one paper [20], DNA analysis from AF was only reported in patents. Despite that, DNA analysis methods have a high accuracy (≈ 100%) and are applicable across several breeds due to the presence of conserved genes. However, although presently the company PLANTegg applies PCR for in ovo sexing (PlantEgg GmbH, Kiel, Germany), its industrial application is restricted because of the lengthy process time (> 60 min) and the consumable costs.

### Immunosensing

Hormones play an essential role in chickens' sexual differentiation, with concentrations changing during incubation and growth of the fetus. A simplified cascade of these main hormones is depicted in Fig. 2. These hormones are mainly gauged via competitive enzyme-linked immunosorbent assay (ELISA) or radioimmunoassay (RIA) due to the high accuracy and sensitivity of these assays. Competitive ELISA or RIA assays are based on the competition between the sample target and the same labeled target for specific antibodies, whereas the label for a target can be either a fluorescent enzyme (ELISA) or radioisotope (RIA). More information about papers and patents dealing with the hormones analyzed using immunoassays is given in Section S2 of Supplementary materials.

The first paper regarding the use of immunoassays dates back to 1979 when Tanabe et al. reported detecting hormones from embryos (d 17 and 20) [32]. Moreover, a paper from Gill et al. [70] showed the possibility of detecting several hormones from d 10 and 14 (Section S2 of Supplementary materials), while showing the hormonal level increase during the incubation stages. Furthermore, in 1998, Phelps protected [29] and published [12] a method measuring estrogens and androgens in extra-embryonic fluid (e.g., AF). Tyczkowski et al. [25] patented another method in 2006, describing an assay type to detect estrogenic steroid compounds using microparticles and a specific fluorescently labeled binding protein.

Furthermore, Butt and Tran [26] patented, in 2002, a new yeast-based assay type for detecting E2 in AF on d 17, claiming higher hormone concentrations in females. Results supporting this patent's claims can be found in a paper from 2010 [71]. However, in 2017, Einspanier [27] protected a method that would allow in ovo sexing based on the E1S concentration in AF extracted from the egg on d 9 and 10. The patent was corroborated by Weissman et al. [ 33], which showed significant concentration differences for E1S at d 9 and E2 at d 10. This scientific report is currently considered a standard golden method for AF extraction and sex determination through hormone concentration measurement in the AF sample.

Some other studies show the presence of hormones in yolk [35, 36] and serum [37]. First, in 2002, Müller et al. [35] studied the relationship between maternal social status and the deposited concentrations of androgens (see Section S2 of Supplementary materials) in freshly laid eggs, concluding that the androgens concentrations in yolk were insufficient for sex differentiation. Second, in 2013, Aslam et al. [36] showed that hormone yolk concentrations were correlated with glucose concentration and unincubated egg dimensions and weight. Finally, Wang et al. [37] showed differences from d 8 to 16, concluding that T, dihydrotestosterone (DHT) and E1 were correlated with sex, and the differences between hormone concentrations varied with the incubation days.

Moreover, a strategy that stands out from the standard ELISA or RIA, while using antibodies, was used by Decuypere and Fey [44], who filed a US patent related to fluorescently labeled antibodies inserted in the egg albumen before incubation. The antibody was specific for several genes encoded proteins from the W-chromosome (e.g., *CHDIW*, *ASW)*, or the Z-chromosome (e.g., *DMRT-1),* or hormones, e.g., anti-Mullerian hormone. However, as discussed in the genetic engineering section, the consumers and industry still question approaches that need the introduction of extra components in consumption products [72].

In summary, the present section showed that d 9 was the earliest day on which it was possible to make this differentiation with E1S (the most promising hormonal biomarker) [33, 69]. Moreover, the company Seleggt (Seleggt GmbH, Cologne, Germany) applies ELISA for in ovo sexing. Considering market application, the 2 challenges for this technology are the considerable cost of ELISA or RIA and the duration of analysis (> 45 min), excluding egg handling and sample extraction.

### Ion mobility spectrometry and mass spectrometry

Analytical techniques such as IMS and MS for hormones and metabolites have also been described as potential in ovo sexing methods. Both techniques require sample vaporization, ionization, and, subsequently, analyte separation based on mass and charge [73]. In 2002, a patent was filed for detecting E2 concentration differences in AF of 5 males and females using IMS [38]. However, the inventors were not certain whether these signals were E2-specific since they could not be related to the fingerprint signal of E2 in a standard solvent (i.e., methanol or acetone). Furthermore, the inventors admitted that the dataset was limited and required more measurements to prove the technique's robustness. Suggested improvements for future experiments were cleaning with a blank solvent between the egg fluid measurements (risking lengthening the analysis time) and combining the IMS with an MS. Usually, coupling of the 2 devices (i.e., hyphenation) is performed to deliver better results for more complex matrices [73].

Next, an MS approach was developed, and the inventors identified a sexual-discriminating, non-proteinogenic metabolite named 3-[(2-aminoethyl) sulfanyl] butanoic acid [39], with higher concentrations in females and more than 90% accuracy on d 9. In combination with another non-specified biomarker, this accuracy improved to more than 95% on d 10. The inventors also recommended using hyphenated systems and thereby managed to analyze a sample in less than 10 s [39]. Next to the DNA analysis and the immunosensing technique, this MS method is one of the three commercial techniques used on d 9 by the company In Ovo (In Ovo B.V., Leiden, The Netherlands).

The analyzed literature shows that IMS can operate with air or nitrogen ambient pressure and temperature. In contrast, the MS operates in a high vacuum and uses special carrier gases such as helium. However, MS instruments are more accurate but also have a higher operational cost [73]. Compared to DNA analysis and immunosensing (see Table 2), IMS and MS do not require sample preparation or incubation, allowing direct sample measurement resulting in a shorter analysis time. These features allow fast acquisition of sex-specific compounds (e.g., hormones or metabolites) in AF of chicken embryos. Furthermore, consumable costs are lower since no primers or antibodies are required compared to the previously described methodologies. An amelioration for the IMS and MS approach would be the investment in scientific research to identify and improve understanding of the measured physiological substances.

### Genetic engineering

There are still several controversies around the use of GMOs for consumption products. Genetic engineering methods modify the animal (or plant) genome by introducing, eliminating, or rearranging specific genes [74]. In the context of in ovo sexing, the primary strategy is detecting a label targeting male embryos' genomes. However, GMOs gather different opinions from country to country. It is possible to classify countries into 1) allowing GMOs, 2) not allowing production but allowing imported GMO products, and 3) blocking both production and import [72]. Section S3 of the Supplementary materials presents a list of these countries.

**Fig. 3:**
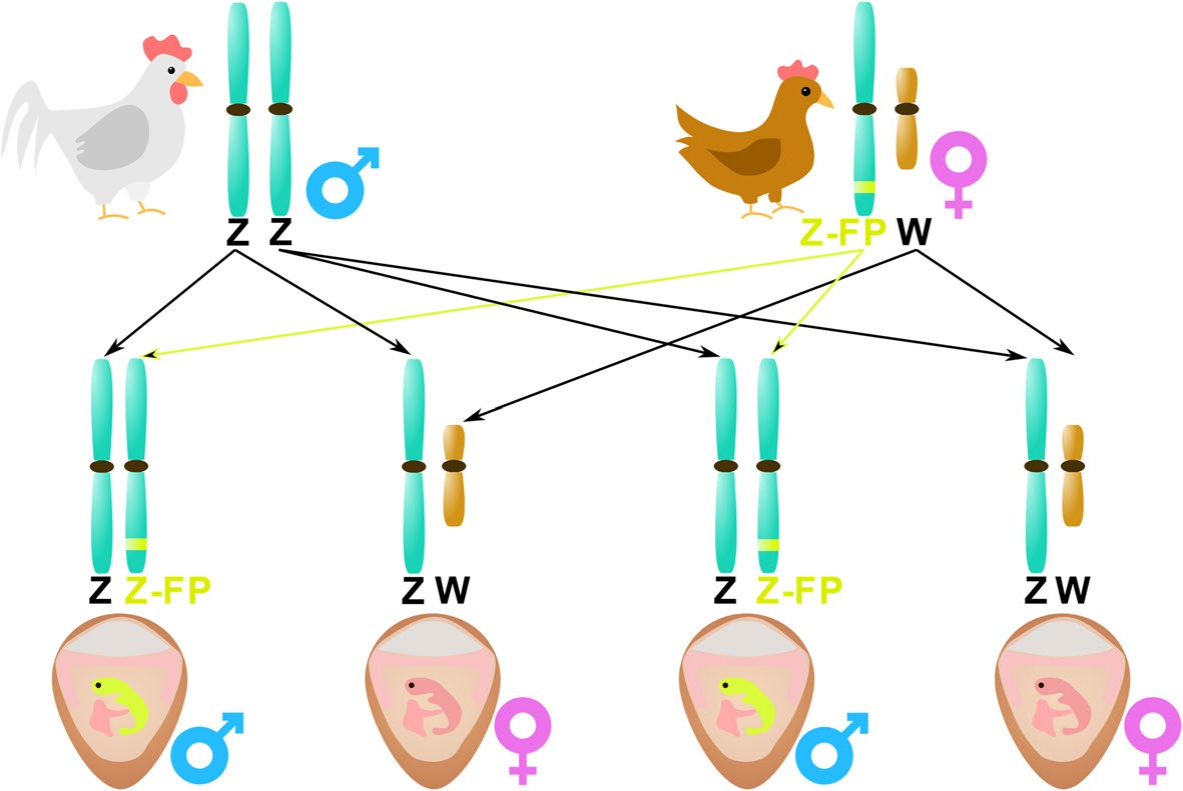
Method for detecting male embryos by introducing a labeled tag. In this example, the labeled tag is a fluorescent protein (FP), in the Z-chromosome of the mother hen (Adapted from [47])

In Doran et al. [47], the fluorescent gene was inserted into the Z-chromosome of the mother hen. The authors performed in vivo transfection of primordial germ cells (PGCs; i.e., gamete precursors showing unique migration activity) into the mother hen at an embryonic stage. Because the target was the mother Z-chromosome, only male embryos from that hen contained the tagged gene, as depicted in Fig. 3. In this context, inventor Offen [45] patented a similar method by the transfection of PGCs with a fluorescently labeled reporter gene. In this case, the PGCs were inserted in the egg on d 2.5, and the reporter gene was incorporated into the embryo genome with clustered regularly interspaced short palindromic repeats and associated protein 9 (CRISPR/Cas9) gRNA (i.e., injected guide RNA) method. This technique is commercialized by the company eggXYt (eggXYt Ltd., Jerusalem, Israel). Data supporting the use of CRISPR/Cas9 can also be found in a study by Lee et al. [48]. Some other techniques were also patented, such as a gene introduction in the broiler hen’s Z-chromosome, leading to the production of a specific protein lethal to the embryos steering to the “natural” death of male embryos [46].

From the available data, only one patent was abandoned until now, whereas the remaining are still active or in the filing phase. This shows that, even though questioned by consumers and prohibited in some countries, GMOs remain a proper alternative to male chick culling [1]. However, a large imbalance exists between the number of patents and scientific papers, with only one paper to date. Hence, more research should be performed on these practices, including the effects of adding fluorescent markers on chickens and consumers. Despite that, as shown in Table 2, genetically engineered cells allow sexual sorting on d 2.5 whilst maintaining an accuracy close to 100%.

### Volatile organic compounds

**Fig. 4:**
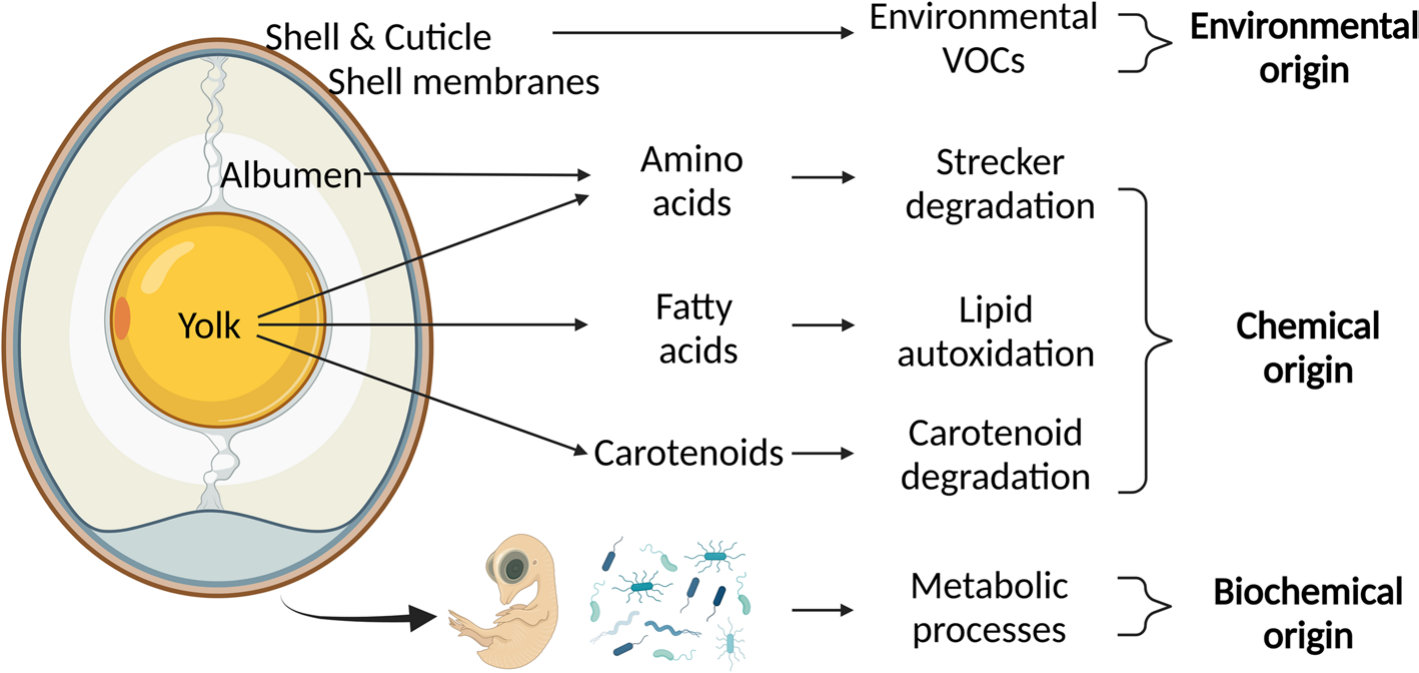
Different possible origins of VOCs. 1) Environmental (shell, cuticle, and membranes can capture VOCs from the environment), 2) Chemical (degradation of amino acids, fatty acids, and carotenoids might result in VOCs), and 3) Biochemical (metabolic processes from the embryo and the microbiota can attribute to an egg’s odor profile) (Adapted from [75]). Figure created with BioRender.com

More recently, researchers also started working on detecting VOCs from avian eggs. These gases are released from the eggshell and can have different origins (e.g., environmental, chemical- or biochemical origins; Fig. 4). Furthermore, it has been demonstrated that the abundance of specific VOCs differs significantly between sexes during incubation [53, 54].

Conventionally, the eggs are enclosed in a recipient to measure these VOCs. After a specific incubation time, extraction is performed by exposing the egg's equilibrated headspace to a sorbent (e.g., solid-phase micro-extraction fiber) or by performing a direct headspace analysis. Relative to the slower gas chromatography-mass spectrometry (GC–MS), faster acquisition techniques are described in the patent literature for measuring egg volatiles, e.g., 1) chemical ionization techniques such as proton transfer reaction-mass spectrometry (PTR-MS), 2) selected ion flow tube-mass spectrometry (SIFT-MS**)** [49], or 3) optical techniques such as Terahertz (THz) spectroscopy [50–52]. The latter optical technique was included in this section since it is used explicitly for VOC sexing. Currently, no VOC in ovo sexing system is commercially available.

VOC in ovo sexing has the potential of non-destructively measuring emitted gases from the egg in an early stage or even on d 0 before the incubation starts (Table 2) [55]. Little is known about the models' robustness and if their accuracies are reproducible. This inherently depends on the limited dataset sizes due to the speed of conventional techniques that analyze at a rate of tens of min to 1 h. Using faster acquisition techniques is expected to allow for more data and faster analysis times (in terms of second), which is also crucial for commercial implementation.

### Other techniques

This section groups different in ovo sexing approaches described in the literature with a unique methodology or without a defined category. Remarkably, the oldest patents were defined within this group, such as measuring magnetic polarity [56–58, 60] or using a galvanometer [59]. Furthermore, only one paper was reported on these pioneering in ovo sexing technologies [63] (although without mentioning the accuracy), based on non-invasive impedance measurements with 4 electrodes on the eggshell of embryos at d 9 of incubation [63]. Regarding the patents, the protection has expired or is inactivated due to non-payment [60].

The following 2 concepts are more recent but did not provide experimental data. The first approach utilized a plaster with a needle inserted through a hole in the eggshell. At the tip of the needle, a fluorescent marker signal could be read once in contact with a sex-specific molecule such as T or E2 [61]. Although the inventors did not specify the marker, it is expected that it might be related to an immunosensing approach. Second, with an electrode close to the shell, a passive recording of the dynamic electromagnetic spectrum on the egg surface would allow sexual sorting prior to incubation. According to the inventors, this electromagnetic field was intrinsically formed through the egg's biological, biochemical, and electrochemical processes [62]. Whereas the patent with the plaster approach was granted, the second one is still under examination. These techniques have not been explored and might require more study. To conclude this section, an overview of all non-optical in ovo sexing techniques is presented in Table 2.

## Optical in ovo sexing methods

This section describes the optical in ovo sexing techniques. Optical techniques are considered to be both physical (i.e., the interaction between the sample and electromagnetic radiation) or geometrical (i.e., imaging) [76]. These approaches are generally non-invasive and allow fast signal acquisition. Although in specific situations (e.g., Raman and fluorescence spectroscopy), a hole must be made through the shell or the membrane to access internal structures inside the egg. Similar to the previous section, Table 3 summarizes the different approaches.

**Table 3:** Overview of optical in ovo sexing techniques

**Category**	**Target**	**Incubation day/ Accuracy/ Throughput**	**Technology (patents)/ Research gaps (papers)**	**Advantages (+) /Drawbacks (-)**	**References**
Raman & fluorescence spectroscopy	Blood, eggshell, germinal disk, or other embryonic structures	d 3.5 to 5 96% accuracy on blood/ d 6 92.3% on embryo and blood/ Low to high throughput depending on the invasiveness	Technology ≅ Research The applicability on blood vessels is well reported in academic literature and is protected by patents Technology > Research A scientific gap exists in the analysis of the eggshell, germinal disk, or other embryonic structures since these are only reported in patent literature	** + ** Early applicable in incubation No liquid or tissue sampling required **-** Intensive sampling procedure and relatively invasive process	**Patents** □ Baron et al. [ 77], ○ Steiner et al. [78], ○ Galli et al. [79], ◊ Schortgen [65], ○ Opitz et al. [80], ○ Popp et al. [81], ○ Schart [82], ◊ Herzog and Hurlin [83], ○ Steiner et al. [84], ○ Hurlin et al. [85] **Papers** Galli et al. [86–88], Preusse et al. [89], Gokdag et al. [90]
IR & THz spectroscopy	Germinal disk	d 0/ Accuracy not defined/ Average throughput	Technology ≅ Research From the same group of authors, both patents and papers are reported for the IR approach Technology > Research Only a patent is reported for the non-invasive THz approach	** + ** Applicable in an early stage **-** IR approach infringes hatchability	**Patents** ○ Steiner et al. [91], ○ May et al. [92], ◊ Xie et al. [93] **Papers** Steiner et al. [94, 95]
VIS–NIR spectroscopy	Heartbeat, blood hemoglobin, movement, feather color, yolk ratio, or growth (opacity)	d 0 to 14 depending on the technology/ High accuracies on color sexing 99%/ Blood sexing or other spectral features range from 80 to 90%/ High throughput	Technology ≅ Research Color sexing in the egg is commercially available and is described in both patent and paper literature Blood detection through the eggshell is described in paper and patent literature Technology > Research Other VIS–NIR spectroscopy on specific egg regions on specific d is only described in patents and lacks scientific background	** + ** Has the potential for non-invasive implementation Equipment is affordable Early application potential **-** Lack of physiological background for sex-determining spectral features described in patents	**Patents** ○ McKay [96], ◊ Hurlin [ 97], ◊ Li et al. [98], ○ Preusse [99], ○ Fujitani [100, 101], □ Rams and Toellner [102], ○ Hebrank [ 103], ◊ Wang et al. [ 104], ◊ Adar [105], ◊ Colvin [106], ○ Ngadi et al. [107, 108], □ Zhao et al. [ 109, 110], ○ Rozenboim and Ben Dor [111], □ Pan et al. [112], ○ Green [113], ○ Zhu [114], ◊ Fischer and Meissner [115, 116], □ Yang et al. [117], ◊ Ngadi et al. [118] **Papers** Göhler et al. [119], Corion et al. [120], Khaliduzzaman et al. [121], Rahman et al. [122], Li et al. [123], Pan et al. [124]
RF & NMR spectroscopy	Gonads, hormones, or metabolites	d 4 to 11 for hormones and metabolites/ accuracy not defined and relatively fast, d 18 gonads/ accuracy not defined and relatively slow	Technology > Research Almost only patent literature has been reported A single research paper reports the difficulty of detecting gonads	** + ** Non-invasive Early incubation **-** No prediction accuracies are reported or known Gonad sexing relatively late	**Patents** ○ Bruins and Stutterheim [40], □ Reynells and Flegal [125], ○ Haase et al. [126], ○ Hergenroder et al. [127], ◊ Sewiolo and Ziroff [128], ○ Jaque Gonzalez et al. [129] **Papers** Davenel et al. [130]
Morphometric studies	Eggshell morphology or blood morphology	d 0 for eggshell/ ~ 93 to 100%/ Relatively fast (using imaging technique), d 4 for blood/ 89.74%/ Relatively high throughput expected, yet equilibrium time needed in a horizontal position	Technology > Research Numerous patents are filed and a few papers One paper is using the formulas from a patent Not all papers did find sexual differences Using the shape index, there was a trend for female eggs to be more spherical, although the overlap was big with male eggs Blood vessel morphology sexing is described both in patent and paper literature, and it might require more research to verify the robustness	** + ** Non-invasive Before or early incubation **-** Not enough scientific evidence that proves this technique works robustly nor an explanation of why there are sexual differences in the egg shape Blood vessel imaging requires a 2 min equilibration time in a horizontal position	**Patents** Taniguchi □ [131], □ [132], ○ [133], □ [134], ◊ [135], ◊ [136], □ [137], □ Lee et al. [138], □ Xiaohui et al. [139], □ Li et al. [140], □ Li [141], ○ Chen et al. [142], ◊ Zhu et al. [143] **Papers** Yilmaz-Dikmen et al. [144], Kayadan et al. [145], Rutkowska et al. [146], Zhu et al. [147]

*d* Day, *IR* Infrared, *THz* Terahertz, *VIS–NIR* Visible-near-infrared, *RF & NMR* Radio-frequency & nuclear magnetic resonance, ○ *Granted*, ◊ *Pending*, □ *Ceased/Rejected/Discontinued*

### Raman and fluorescence spectroscopy

Generally, Raman spectroscopy is used after excitation with a monochromatic laser, having identical energy photons to analyze the frequencies of scattered radiation, obtain information about molecule energy levels, and study molecular vibrations and rotations. The broader fluorescence background intensity is directly re-emitted energy [148]. In ovo sexing Raman spectroscopy has been applied in an invasive and non-invasive way. The former analyzes the hemoglobin content in blood vessels [86] or germinal disk cells [149], and the latter focuses on sex hormones or other sex-related compounds in the eggshell [77]. Both methods are further explained in this section.

**Fig. 5:**
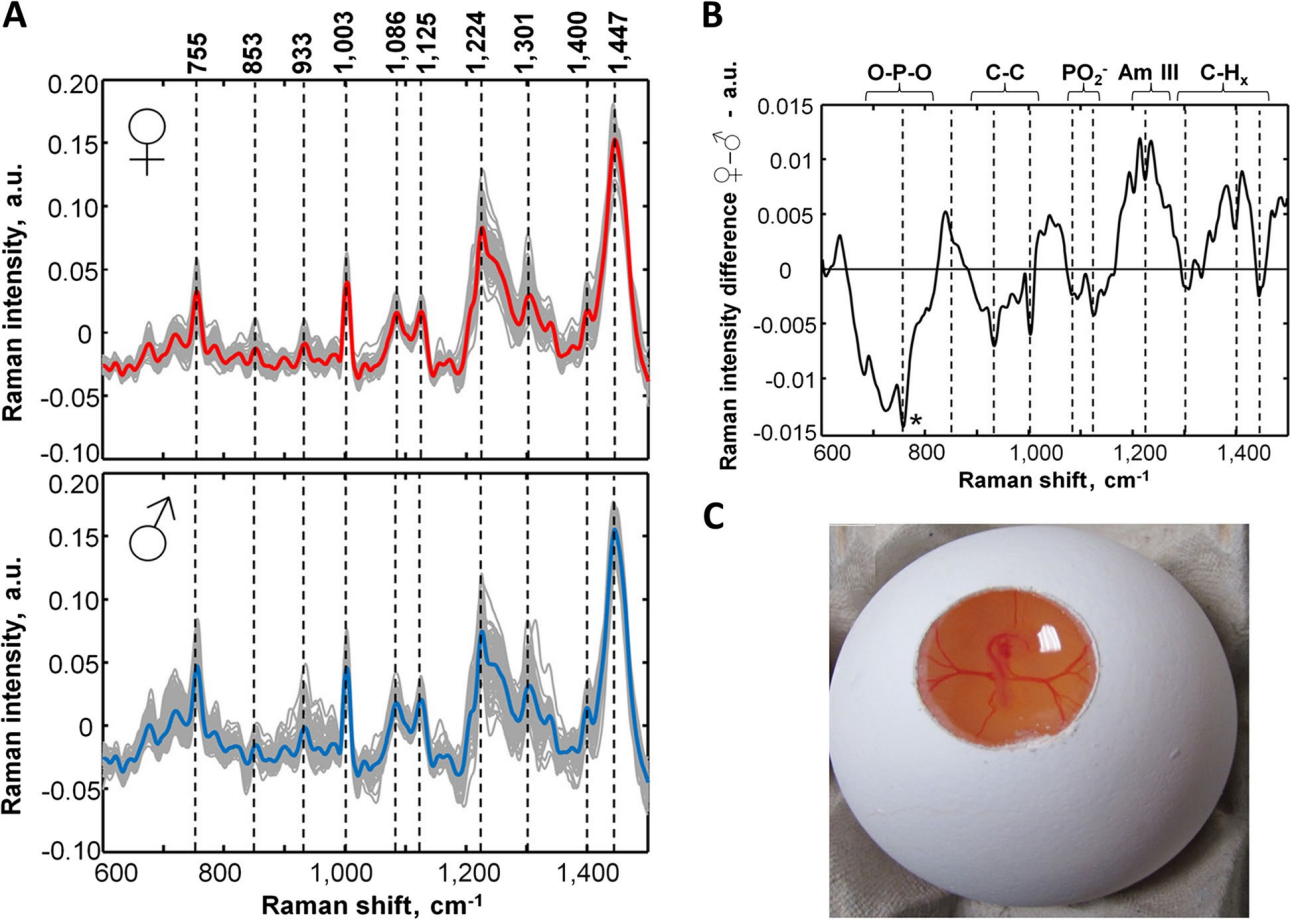
Raman spectroscopic in ovo sexing on d 3.5. **A** Preprocessed Raman spectra with mean female spectrum on top (red color) and mean male spectrum at the bottom (blue color) with the most prominent bands being indicated. **B** The mean male spectrum was subtracted from the mean female spectrum indicating the main spectral regions. **C** Opened shell to access the embryo's blood vessels [88]. The figure is reprinted with permission from ACS Publications

An invasive Raman and fluorescence spectroscopy in ovo sexing method on d 3.5 was developed by Galli and co-authors and described in both patents and papers (Fig. 5) [78, 79, 86–89, 150, 151]. With this in ovo sexing method, the laser excited a target (i.e., an extraembryonic blood vessel) and Raman bands with differences in blood chemistry and/or a higher fluorescence signal were observed in males due to a higher hemoglobin content (Fig. 5A and B).

In contrast to the previous technique, non-invasive Raman spectroscopy approaches are solely described in the patent literature [65, 77]. The inventors claimed that sexually-discriminating substances such as sex-related genes, gene products, or sex hormones are spread throughout the egg and migrate to the eggshell. Subsequently, a focal point was set for a monochromatic laser to excite molecules inside or near the eggshell. Next, the reflected Raman signal was captured, and the spectral response would provide information on fertility and sex [65]. However, no experimental data was provided on which substances were measured, on which incubation d it took place, or what the classification rate would be.

In another application, the detection of germinal disk cells' autofluorescence decay using a fluorescence spectrometer was described with a relatively low prediction accuracy (≈ 75%) [86]. Similarly, fluorescence excitation of blood vessels, germinal disk cells, or other embryonic structures was stated with accuracies ranging from 84.7% to 92.3% on d 3 to 6, respectively [152]. Another application suggested using Raman spectroscopy to measure the germinal disk cells using, for example, feather pulp from which a model was built with a 95.4% accuracy [81, 149]. Finally, all these technologies required access through the eggshell to target the embryonic structures. This is considered an invasive approach that risks impacting embryonic development. Nevertheless, this technology can potentially be used early in the incubation process.

Despite the early detection and decent accuracy of 96%, the intensive sampling procedure by windowing the eggshell (Fig. 5C) and the sub-optimal analysis speed of 40 s/egg (see Table 3) might explain why this technology has not been commercialized yet, even though it has been acquired and further developed by Agri Advanced Technologies (AAT GmbH, Visbek, Germany) [86]. The company Seleggt, which was earlier mentioned for its immunosensing approach, also invented an approach in which small holes are laser-cut through the eggshell that can be accessed by a laser beam for excitement and spectrum acquisition of a blood vessel. Furthermore, they envisioned an embodiment in which an extra-embryonic blood vessel is laser-cut and spectroscopic analysis of the expelled blood is performed on the exterior of the egg. The blood is expelled by applying pressure to the egg’s interior using a pressure cup on the blunt side [153]. By expelling blood from the egg, this novel method could make it easier to obtain blood samples and create new possibilities for utilizing spectroscopic analysis. However, it should be noted that this manipulation poses also a risk of contamination of the embryo.

### Infrared and Terahertz spectroscopy

Infrared spectroscopy (IR) sexing is based on the difference in DNA content between sexes, whereby the males have approximately 2% more DNA [154]. These differences were detectable through an overall higher absorbance by male blastoderm cells extracted from the germinal disk, placed on an attenuated total reflection crystal [94]. Like the Raman spectroscopic approach, IR can retrieve sex-specific information in a very early incubation stage (≤ d 0). This approach was applied even before incubation using Fourier-transform IR by Steiner et al. [91, 94, 95]. Towards industrial application, the inventors integrated this crystal inside a probe with a cone-shaped tip that could be inserted through a hole in the eggshell [91]. Due to the earlier-mentioned risk for the egg, the inventors stated in another paper that this method could not be further exploited [86]. Nevertheless, the US patent is still active [91]. A more recent patent presented the application of IR reflectance to determine the sex of an intact egg by analyzing its surface. While the inventors acknowledged the significance of avoiding the measurement of deeper layers to prevent water absorbance, they did not provide any specifics about the biomarkers being targeted, the accuracy of the predictions, or the timing of sex determination [93].

Similar to the IR spectroscopic technique, a THz spectroscopic approach was described, claiming that the waves would pass the intact shell and cause molecular vibrations of sex-specific DNA structures in germinal disk cells [92]. THz wavebands are situated next to IR waves and can be absorbed by DNA molecules, thereby providing specific signatures [155]. The patent for this THz approach has been granted, although no experimental data were reported.

In summary, although this early application holds commercialization potential, the process is relatively destructive, i.e., a small hole for probe insertion has to be made, increases contamination risks, and might affect hatchability, as shown in Table 3.

### Visible-near-infrared spectroscopy

Visible-near-infrared (VIS–NIR) spectroscopy has the widest range of targets described for in ovo sexing. This non-destructive technique measures vibrations caused by the stretching and bending of hydrogen bonds with carbon, oxygen, and nitrogen. Furthermore, it is widely used for assessing agricultural product quality and has the advantage of being fast, non-invasive, economical, and accurate [156]. Classically, VIS–NIR spectroscopy consists of a single spectral dimension and can be extended by 2 spatial dimensions (i.e., *X* and *Y*), resulting in a 3-dimensional hyperspectral image [157], and enabling focusing on a specific region of interest within the egg. The targets described in the state-of-the-art vary in 1) sex-related blood absorption [99, 100, 122], 2) heart rate or body movement [102, 103, 105, 158], 3) egg yolk ratio [101], 4) egg photoluminescence [106], 5) embryonic growth rate [121], 6) sexual-specific coloring [96–98, 119, 120], and 7) sex determining spectral features on the germinal disk or other regions in the egg [107–118, 123].

**Fig. 6:**
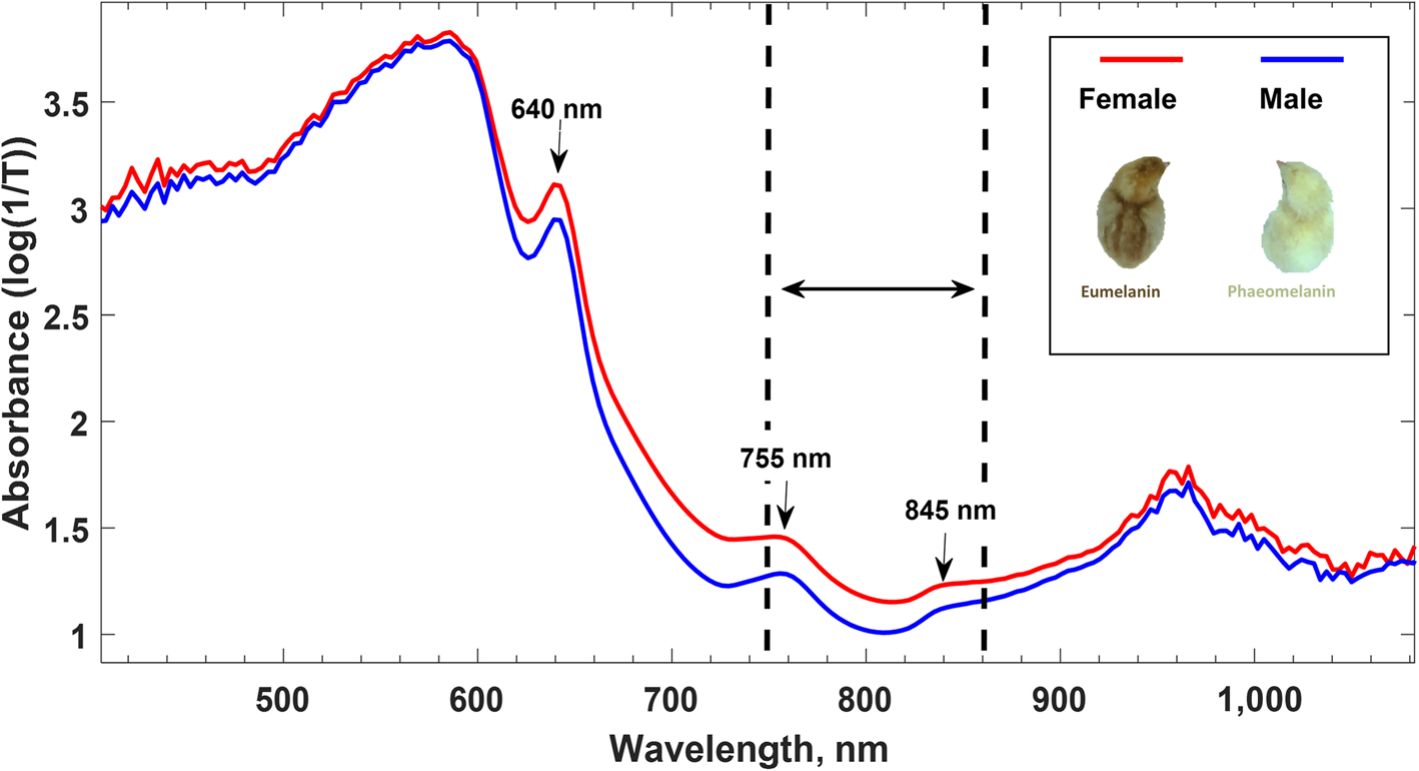
Melanin vs. phaeomelanin absorption on incubation d 14 using VIS–NIR. At indicated wavelengths, females (red line) with eumelanin pigmentation have a higher light absorption (i.e., the logarithm of the inverse of the transmission). The selected wavelength range indicates the relevant window for sexual determination (Adapted from [160])

In general, the techniques described in the patent literature for the aforementioned distinct targets 1 to 4 did not define accuracy or the specific day for performing sexing. The egg opacity was used to measure embryonic growth, reporting an 84% prediction accuracy from d 16 to 18 [121]. Furthermore, VIS–NIR transmission measurements on eggs from chicken breeds with a sex-specific brown feather coloring is a well-established method being commercially applied by Agri Advanced Technologies (AAT GmbH, Visbek, Germany) on d 13 to 14 with an accuracy of up to 99% in less than a s per egg [96, 97, 119, 120]. The more brown pigmentation in females (i.e., eumelanin) relative to the more yellow males (i.e., phaeomelanin) resulted in higher absorption of light in female embryos (Fig. 6). Similarly, a sex-linked inhibitor of dermal melanin located on the Z chromosome determines shank pigmentation in chickens [159]. This trait was used by inventors for non-invasive in ovo sexing on incubation d 13 to distinguish dark-shanked females from bright-shanked males, claiming a theoretical 99.99% prediction accuracy [98].

Earlier application of VIS–NIR was described for blood absorption on d 0 to 7 [ 99, 100, 122]. Without explicitly mentioning accuracies, Rahman et al. [122] found a significantly higher hemoglobin absorbance in males on d 3 (*P* < 0.001). In contrast, a higher hemoglobin absorbance was found in females on d 7 and was hypothesized to be a result of a temporary coupled effect of estrogen receptor synthesis and enzymes on erythropoiesis (*P* < 0.05).

Finally, multiple hyperspectral imaging approaches were described in the patent literature wherein spectral features of selected regions within the egg were used to determine the sex (e.g., the middle region or the germinal disc). The highest reported accuracies were 100% on d 0 [161], 82.86% on d 10 [112], and 80% on d 12 [111]. Especially for the d 0 models, the reproducibility should be investigated further since the inventors did not divide the dataset into a test set and a separate validation set for model robustness.

As summarized in Table 3, the main advantages of VIS–NIR in ovo sexing are the relatively fast signal acquisition time, potential for non-invasive application, and absence of consumables. Generally, it has a good balance between scientific and industrial reporting. However, sometimes there is a lack of physiological background on the spectral features to prove validation and guarantee the robustness of statistical models.

### Radio-frequency and nuclear magnetic resonance spectroscopy

Radio-frequency (RF) spectroscopy involves the use of electromagnetic waves in the RF range to study the characteristics of materials, including atoms, molecules, and other substances [162]. One patent described the emission of an electromagnetic wave frequency between 30 kHz and 67 GHz toward the egg, followed by capturing the reflected signal for sex determination [129]. Nuclear magnetic resonance (NMR) is another suggested technology that uses RF waves to induce an external magnetic field and observes the resonant absorption of energy by the excited nuclei [163]. A first method was patented in which embryos were imaged using magnetic resonance imaging (MRI) during the transfer from setter to hatcher [125]. Here, the processed images revealed the sex by visualizing 2 testes in males and one ovarium in females. However, Davenel and co-researchers [130] failed to identify gonads since these develop on top of the kidneys, and the MRI could not distinguish between both tissues. Furthermore, they questioned the applicability at industrial speed since gonad imaging using MRI was relatively slow. Nevertheless, this concept is commercially applied since January 2023 on d 12 and is further optimized by Orbem (Orbem GmbH, Munich, Germany). Per device, the company claims to have a processing speed of 3,000 eggs per hour and a theoretical prediction accuracy of 98% [164].

In the current patent utilized by Orbem [126], the inventors defined 3 NMR parameters in which they claimed to determine the sex starting from d 5. These NMR signals' specific origin was unknown and assumed to be a chemical shift of metabolites or hormones. This technology is under development for commercialization by Orbem [164]. Concerning the detection of embryonic metabolites or hormones, other non-imaging NMR solutions were reported in patent literature. One non-invasive method for metabolites was described on d 4, where the ratio between nucleobases from free-floating DNA relative to the abundance of aromatic amino acids in blood was significantly lower for males [127]. Further, an invasive NMR method on freeze-dried AF from d 9 to 11 on metabolites (i.e., choline, valine, and glucose) was reported by Bruins and Stutterheim [40]. Finally, a non-invasive approach in which differences in E2 and T would be measured through NMR was claimed [128]. No scientific literature has reported metabolite or hormone detection for in ovo sexing with NMR.

The main advantage of NMR is its potential for non-invasive analysis (see Table 3). The moment of application depends on the target and is more in favor when early applicable. On the other hand, this approach has long acquisition times and requires more scientific reporting, wherein the sex-specific features are studied more deeply.

### Morphometric studies

In the following category, egg and embryo morphometric studies were considered since both use images from industrial cameras. The first approach was patented by Taniguchi, who published different applications from 2001 to 2022 in which the egg shape was used to determine the sex [131–136]. Similarly, Lee et al. proposed an egg-shape-based sexing method, but this patent was ultimately discontinued [138]. Although applicable before incubation, sex differences in egg dimensions are likely to be minimal, and ratios of these parameters will be required to improve the accuracy. The described concept relied on the idea that male eggs would generally be less spherical and more pointed than female eggs [131]. Images were used to measure the shape by quantifying the egg length, width, and distance from the equator to the blunt end. The protection for this patent expired in 2019, and more recently, a follow-up invention included egg distortion [136].

Concerning the concept of the more spherical-shaped female eggs and more pointed male eggs, similar indications have been published in a study on white-layer eggs [144]. The researchers measured the width and length and calculated a shape index that would discriminate the sex from this. However, the measured overlap between the sexes was considerable and did not allow a highly accurate sexual separation. Kayadan and Uzun [145] used a similar strategy and showed that using the egg shape index they could predict female embryos with 80% and males with 81% accuracy.

**Fig. 7:**
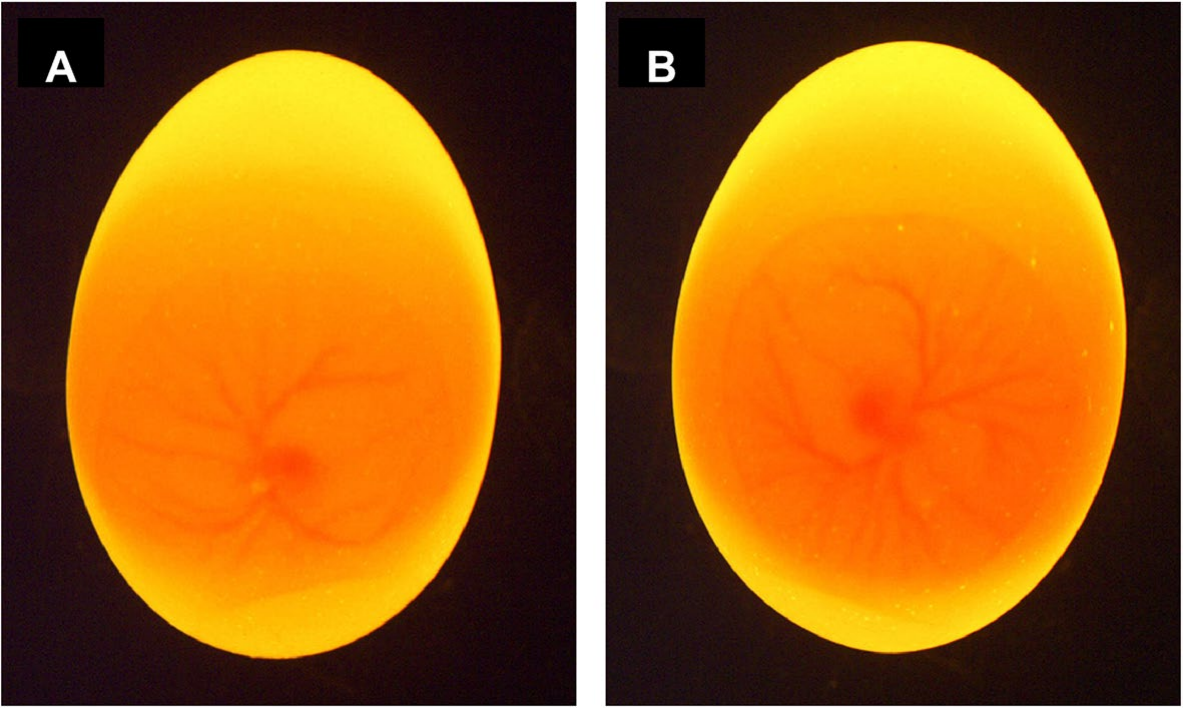
Machine vision images depicting eggs containing embryos on d 4. **A** Female individual with intuitively thinner main blood vessels and fewer lateral vessels with an asymmetrical distribution. **B** Male individual with thick main blood vessels and multiple lateral vessels with a symmetrical distribution (Adapted from [147] with permission from Elsevier)

The second approach used machine vision imaging of embryonic blood vessels around d 4 [139–143, 147]. This technique is assumed to be low-cost and requires a light source, an industrial camera, and a computer. After egg illumination, an asymmetry in the blood vessel branching was observed for the female embryos and was linked to the asymmetric gonadal development in which the right gonad regresses, and less blood is assigned (Fig. 7A). On the other hand, the blood vessel branching would be symmetric for the equally developing testes in the males (Fig. 7B). The authors obtained an accuracy of 89.74% on the prediction set using white eggs [147]. However, it is questioned whether this technique would also work for brown eggs, given that the brown shell might absorb more light and complicate the blood vessels' visualization through the shell.

This approach received increased interest due to the current higher performance of cameras and software for image analysis and has been explored to study eggshells non-invasively and blood vessel morphometry, enabling fast screening. Advantageous is that the first method was applicable before incubation, whereas the second method required blood vessel development in early incubation. However, whether the prediction accuracy is high enough to use morphometric differences between sexes is questioned. Nevertheless, the technique is described in both patents and papers and is expected to hold potential for early and non-invasive in ovo sexing. An overview of all the optical in ovo sexing techniques is presented in Table 3.

## Trends of in ovo sexing techniques

### Commercialized in ovo sexing techniques

**Fig. 8:**
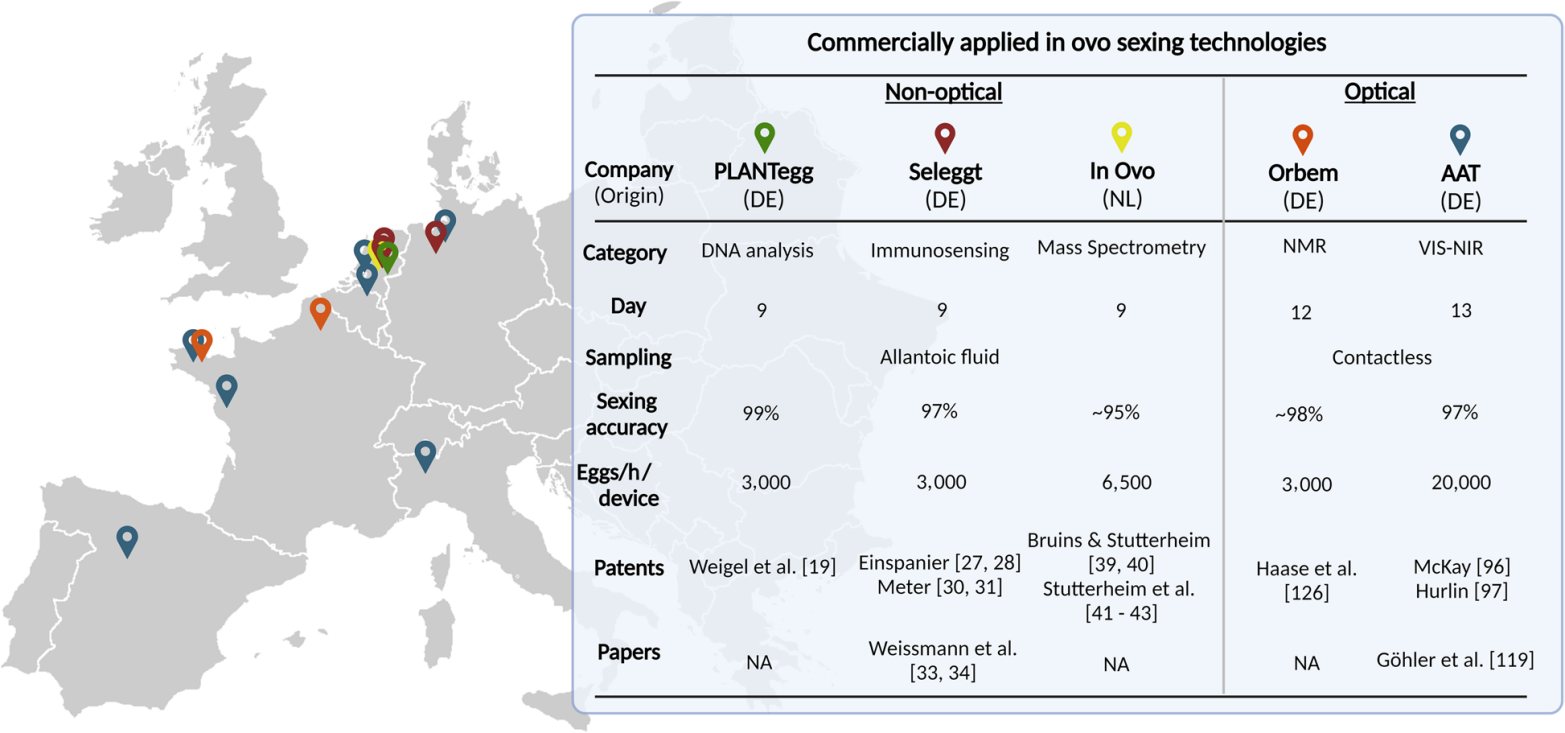
Geographical distribution and main characteristics of the 5 commercialized in ovo sexing techniques. Four companies are German (DE), whereas one company is Dutch (NL). The non-optical techniques are applied in the Netherlands and Germany, the NMR technique is applied in France, and the VIS–NIR technique is applied in Spain, Italy, France, Belgium, the Netherlands and Germany [164, 166–169]. The prediction accuracies indicated by ~ are claimed by the companies and may necessitate additional validation of their robustness through field implementation (figure made with BioRender.com)

Out of the 11 different in ovo sexing categories, 5 approaches are proven to be industrially applicable and are currently commercially operational in West Europe. An overview of these techniques together with their geographical distribution and main characteristics is presented in Fig. 8. The 3 non-optical techniques have in common that they require allantoic fluid sampling [41, 43], whereas the 2 optical techniques can be performed contactless. The sexing accuracies vary between 95% and 99% and the throughput per device ranges between 3,000 to 20,000 eggs/h. It is noted that the optical techniques require an embryo of 12 to 13 d old to be applicable, relative to the 9 d for the non-optical techniques. Based on the latest research on pain perception, it has been suggested that embryos may begin to experience pain as early as d 13 [10]. This implies that the AAT method would be the only approach in situations where embryos may be capable of experiencing pain. As a solution to this, the company has developed a stunning approach for incubation eggs [165]. While each of the 5 approaches exhibits commercial feasibility, none of them satisfies all the requisite features for achieving complete success. It is expected that the approach with the highest cost-efficiency will emerge as the most viable option for sustainable success. Finally, AAT has at present the widest-distributed in ovo sexing technique (Fig. 8). This is possibly explained by its relatively low investment cost relative to the other companies. Nevertheless, their approach is only applicable to breeds exhibiting sex-specific brown feather coloring and it is anticipated that at least one of the other technologies will cover the market for the other breeds.

### Geographical distribution of techniques

**Fig. 9:**
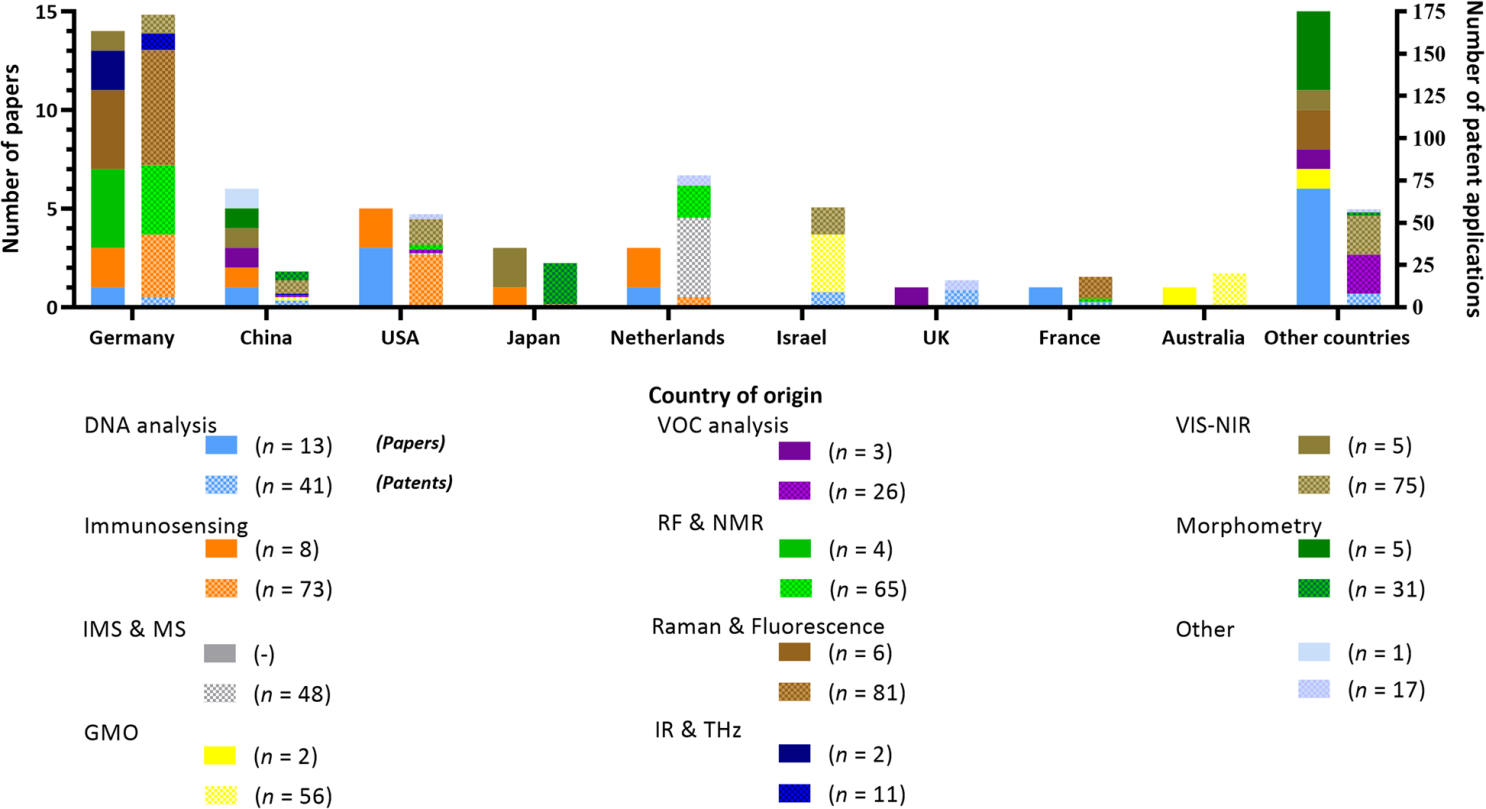
Geographical distribution of papers and patents on in ovo sexing in countries of origin. Per country, the first and second bar consist of the number of paper (plane colors) or patent applications (dotted colors). The “other countries” contain countries in which only a single paper or a limited number of patents was published. Concerning the papers, these were: India, Italy, Sweden, Belgium, and Poland. For the patents, these countries were: Korea, Ireland, Switzerland, Malta, Canada, Romania, Russia, and Belarus

The geographical distribution of the in ovo sexing techniques reported in papers and patent applications by origin is presented in Fig. 9. Similar to the dominating presence of German companies (4 out of 5 in Fig. 8), it was observed that Germany had, by far, the majority of patent applications (*n* = 173) and the largest number of papers (*n* = 14). Germany was the first to ban male chicken culling within the poultry industry [5], potentially explaining the higher pressure to find new solutions and the high number of papers and patent applications. Furthermore, the apparent dominance of Germany in companies, published papers, and patent applications suggests that Germany played a leading role in pushing to ban the current global practice of male-chick culling. Next to Germany, the Netherlands (*n* = 78), and the USA (*n* = 73), were the countries with the highest number of patent applications, accounting for 62% (*n* = 324) of the overall number. These numbers were not necessarily at the same level as the number of papers. Overall in most countries, there are more patent applications than papers. Putatively, this was linked to the male culling problem requiring an industrial solution. Subsequently, researchers would prefer to protect their approach as soon as possible via a patent before disclosing their work through a paper. An extreme example was Israel, which had 59 patent applications and no papers.

To further elaborate on the current poultry market shares between countries, China leads the list on egg production, followed by the US, Europe, and India [170]. The trend in egg production was positively correlated with the seek for protection for the US, China, and Germany (the latter being the biggest egg producer in the EU; Section S4 of the Supplementary material). Finally, it was observed that the University of Dresden in Germany was the main publisher, with 8 papers, while In Ovo (In Ovo B.V., Leiden, The Netherlands) was the main patent applicant with 9 patent families. The Supplementary materials present these data in Sections S5 and S6, respectively. Next to the immunosensing approach of the University of Leipzig and the company Seleggt, German researchers seem to predominantly work on optical in ovo sexing techniques.

### Publication trends of papers and patents over time

**Fig. 10:**
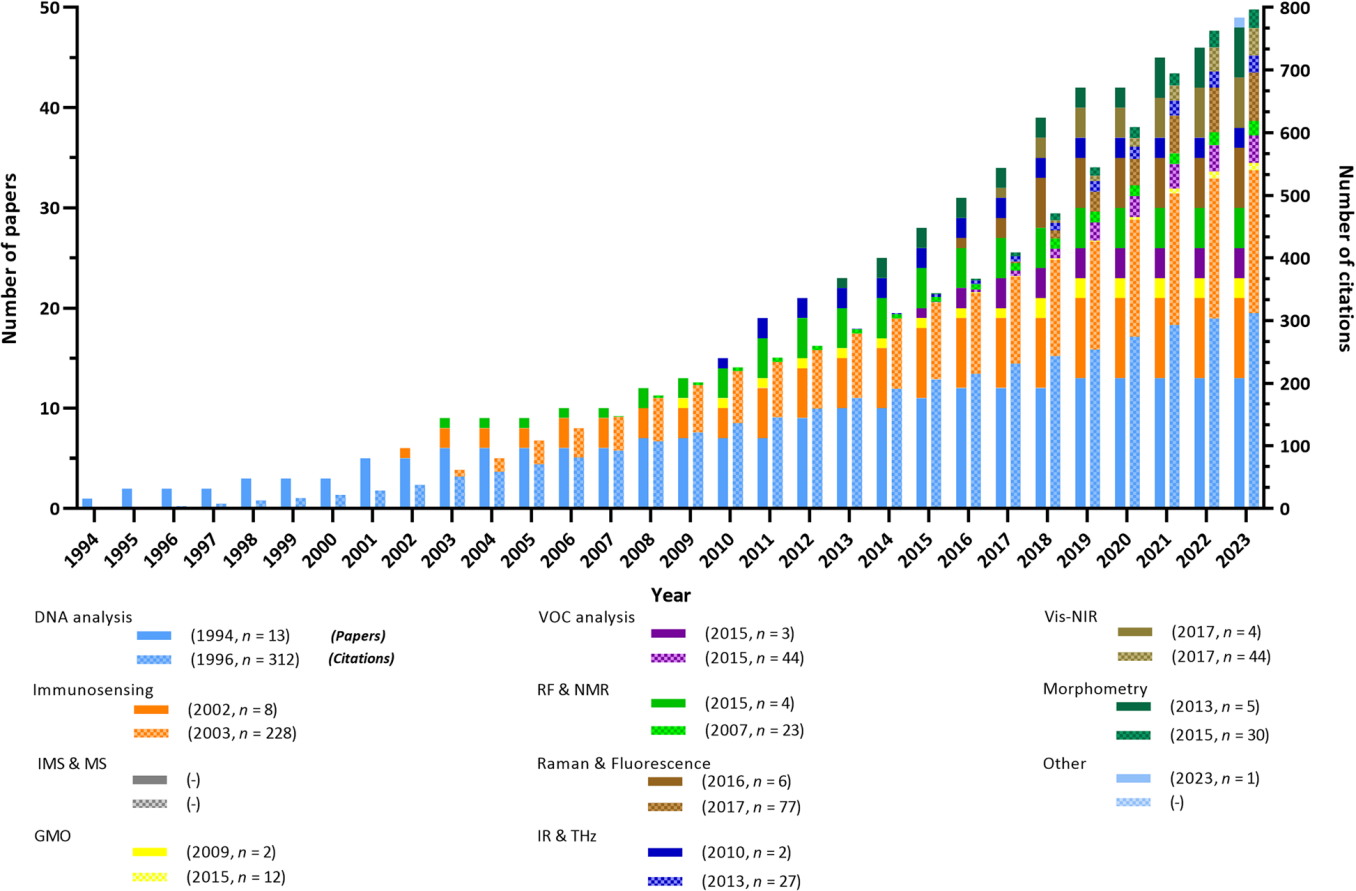
Cumulative growth of the number of in ovo sexing papers (plane colors) and citations (doted colors) until 2023. For each category, the first publication and citation year and the total publication and citation number by 2023 are presented in between parentheses. In total, 49 papers and 798 citations on in ovo sexing were reported until 2023, included

Next, a quantitative representation of scientific publications and patent applications was organized in infographics to better understand the evolution of in ovo sexing technologies over time. Figure 10 shows the scientific literature's growth and citations over time for the different categories. Since the first scientific publication in 1994, constant paper and citation growth has generally been observed for all techniques, with a total of 49 publications and 797 citations by May 2023. No exponential growth was observed for any category, probably because no technology sufficed all the necessary market requirements. As of 2023, DNA analysis was the category having accumulated the most scientific publications (*n* = 13), followed by immunosensing (*n* = 8). Perhaps this is due to the fact that these technologies were the first to be reported and that they are usually applied as reference techniques. Similarly, their considerable influence on the field was confirmed by the highest citation number for the DNA analysis category (*n* = 312), followed by immunosensing (*n* = 228). In contrast, the IR & THz and GMO categories were the least reported techniques (each with 2 papers), with the least number of citations for the GMO category (*n* = 12). The IMS and MS category had no reported scientific literature. An overview of the journals with two or more publications on in ovo sexing is presented in Section S7 of the Supplementary materials.

**Fig. 11:**
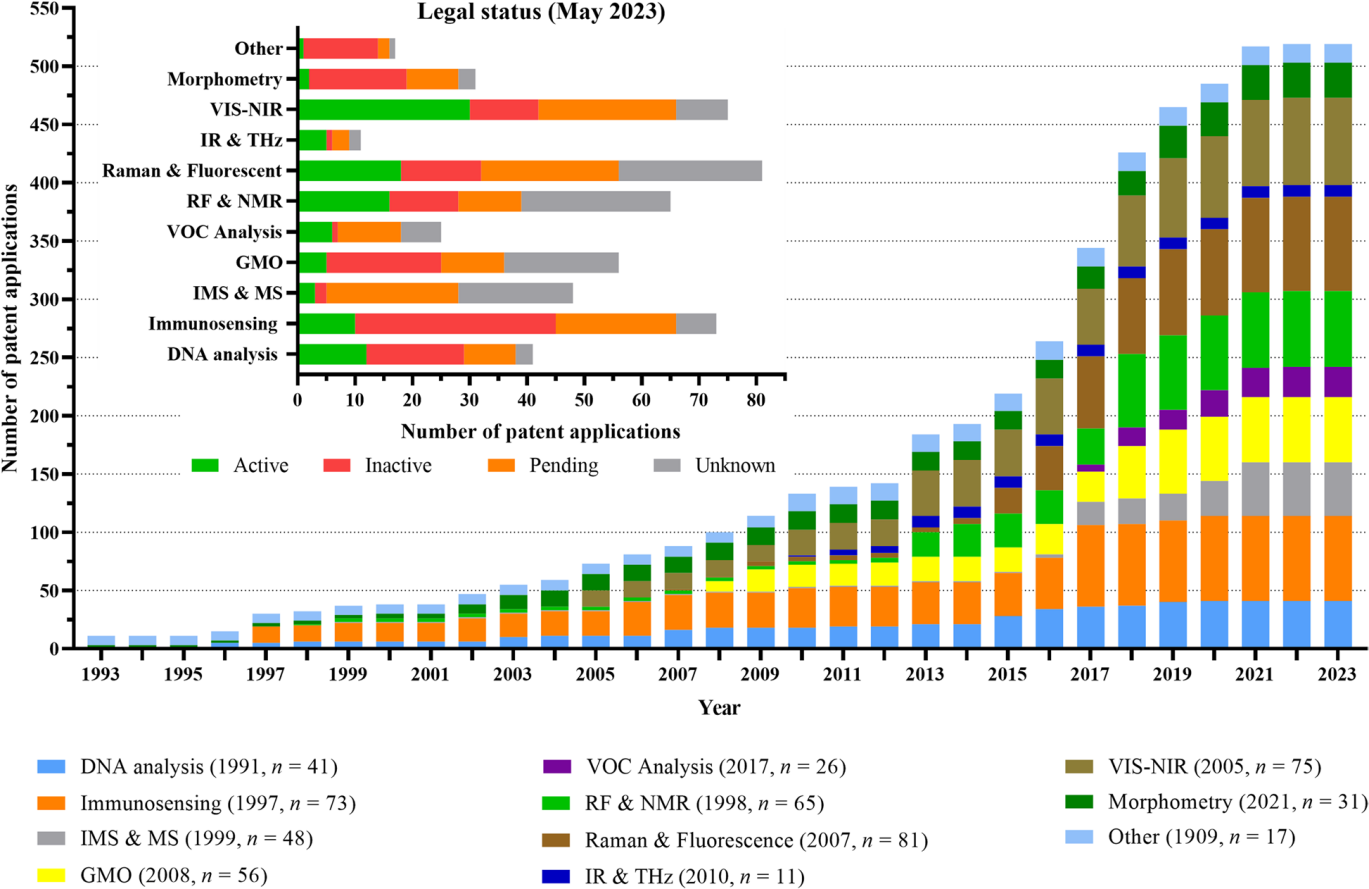
Cumulative growth of the number of in ovo sexing patent applications until 2023. For each category, the first publication year and the total publication number in 2023 are presented in between parentheses. In total, 115 unique patent families and 524 patent application numbers were registered until 2023. The legal status (active, inactive, pending, or unknown) of the patents on May 2023 for each category is shown in detail in the graph

Furthermore, Fig. 11 visualizes the growth of the patent application numbers over the years and the legal status (active, inactive, pending or unknown) of the patents on May 2023. The original dataset can be found in Section S8 of the Supplementary materials. In total, 115 patent families have been found, resulting in 524 patent application numbers (multiple patent application numbers can be registered for one patent family if a patent has to be protected in multiple regions). The interest in a specific category over time was analyzed considering the application number evolution throughout the years. Similar to Fig. 10, the timeline started in 1993 for representation’s sake. Prior to 1993, 8 patents have been filed from the "other" category with the first one dating from 1909 for measuring magnetic polarity. Furthermore, 2 other morphometry-related patents and one DNA analysis-related patent were filed before 1993. For more details, reference is made to the patent dataset in the Section S8 of the Supplementary materials. Since the oldest patents originate from the “other” category and morphometry, a significant number of patents in these categories have become inactive as a result of expiration.

Steady growth with sporadic peaks was observed for specific technologies, e.g., between 2014 and 2018, Raman and fluorescence-related patents increased from 5 to 65. Some other examples of noteworthy increases were from 2012 to 2023: GMO (20 to 36), RF & NMR (3 to 62), and VIS–NIR (23 to 52). Interestingly, these exponential growths were observed for some of the most recent techniques. The first GMO patents dated from 2008, starting with 9 patent applications, and VOC analysis from 2017, starting with 6. Since VOC analysis is a relatively new approach to in ovo sexing, a small number of patents are inactive (*n* = 1), while a larger portion is still pending grant approval (*n* = 11). This trend is also observed in the IMS & MS category, where a relatively high number of patents are awaiting patent issuance (*n* = 23). This observation indicates their potential growth. Overall, active patents across all categories indicate that investments are being made in all approaches without any discrimination.

In 2023, the category with the most patents was the category of Raman and fluorescence spectroscopy (*n* = 81). The category with the least patents was IR & THz (*n* = 11). Considerable investment has been put into Raman and fluorescence spectroscopy since it can perform sexual detection on d 3.5 with high accuracy, which is an advantage for animal welfare (i.e., before the pain onset) and for the industry (i.e., the rapid replacement for female eggs). This technology is also being developed by AAT entitled “early spectroscopic method” [169]. However, it did not reach the market yet and this is potentially due to the challenges associated with effectively automating the intensive sampling process, reaching satisfying levels of prediction accuracies, and mitigating adverse effects on hatchability.

By comparing Fig. 10 and Fig. 11, it became apparent that most of the technologies were first protected with patents before being published in scientific papers. Some examples of this were seen with DNA analysis, in which the first patent application was filed in 1991, while the first paper dates to 1995, or even the Raman spectroscopic approach for which the first patent application dates to 2007, while only being reported in papers since 2016. In some cases, e.g. IMS and MS, there were no published papers, only patent applications filed in 1999. Exceptionally, the VOC analysis approach was first reported in a paper in 2015, whereas its first patent application is from 2017, being the most recently patented technology. Finally, the growth trends between papers and patents tended to be different, with the latter looking similar to an exponential curve until 2017 when the growth stabilized until 2021. The different growth rates led to ten times more patent applications than papers. This could be explained by the number of different patents (*n* = 524) filed under the same family number (*n* = 115).

## Conclusion

Since 1907, in ovo sexing technologies have been developed to tackle the issue of male chick culling in the poultry industry. This review shows the effort that has been put into developing in ovo sexing technologies, enabling sex determination before hatching. Until now, all the reported technologies (both in patents and papers) do not satisfy the market requirements or create other problems, such as environmental or new ethical issues. Based on this review, no clear consensus can yet be reached on which technology is best suited for in ovo sexing, which is mirrored by the newly emerging techniques. Among all the presented technologies, we considered the optical techniques promising since they offer the potential for non-invasive and high throughput screening. However, most optical techniques fail to meet the accuracy or incubation time requirements. An exception is Raman spectroscopy, an invasive optical technique with the desired early detection (d 3.5). However, more improvements are needed to make it less invasive, and guarantee unaffected hatchability.

Furthermore, the technology that most recently emerged in patents was the VOC analysis approach. The potential of being non-invasive and early applicable has been demonstrated in papers. However, it requires more research to robustly define the relevant biomarkers and detect them with fast equipment. Other standard technologies, such as DNA analysis, immunosensing, or even mass spectrometry, are well-known analyses used across different applications and will most likely continue to be used for in ovo sexing.

However, to finally succeed in placing a technology in the industrial setting, it is first necessary to obtain harmony between industry, academia, and governments to decide on the best approach and requirements to solve male day-old chick culling. Although none of the 5 companies currently satisfies all the requirements for complete success, their solutions provide valuable insights into the potential and expectations for in ovo sexing applications in commercial hatcheries. As such, their performance in the market should be closely monitored and studied.

## Supplementary Information

**Additional file 1: S1.** Search methods and keys.** S2.** Papers and patents related to immunosensing.** S3.** Countries allowing or banning GMO importation.** S4.** Patent application numbers.** S5** Distribution of papers per publishing institutions.** S6.** Applicants with active patent filing.** S7.** Top journals and individuals.

**Additional file 2: S8.** Papers and patents dataset.

## Data Availability

The paper and patent dataset generated for this review is available in an Excel file in the Supplementary materials Section S8.

## Abbreviations

A4: Androstenedione
AF: Allantoic fluid
CHD: Chromodomain helicase DNA
CRISPR/Cas9: Clustered regularly interspaced short palindromic repeats and associated protein 9
DHT: Dihydrotestosterone
DMRT-1: Doublesex and mab-3 related transcription factor 1
E1: Estrone
E1S: Estrone sulfate
E2: Estradiol
ELISA: Enzyme-linked immunosorbent assay
FRET: Fluorescence resonance energy transfer
GC–MS: Gas chromatography-mass spectrometry
gDNA: Genomic DNA
GMO: Genetic modified organisms
IMS: Ion mobility spectrometry
IR: Infrared
MRI: Magnetic resonance imaging
MS: Mass spectrometry
NMR: Nuclear magnetic resonance
P4: Progesterone
PCR: Polymerase chain reaction
PGCs: Primordial germ cells
PTR-MS: Proton transfer reaction-mass spectrometry
qPCR: Quantitative PCR
RF: Radio-frequency
RIA: Radioimmunoassay
SIFT-MS: Selected ion flow tube-mass spectrometry
SPME-GS-MS: Solid phase micro extraction-gas chromatography-mass spectrometry
T: Testosterone
THz: Terahertz
VIS–NIR: Visible-near-infrared
VOC: Volatile organic compound
WPKCI: W-protein kinase C iota
