# Supplementary material for: Trends in in ovo sexing technologies: insights and interpretation from papers and patents
Source: J Anim Sci Biotechnol. 2023 Jul 14;14:102. doi: 10.1186/s40104-023-00898-1 (PMC10347793; doi:10.1186/s40104-023-00898-1)
Supplement: Supplementary file 1 — Additional file 1: S1. Search methods and keys. S2. Papers and patents related to immunosensing. S3. Countries allowing or banning GMO importation. S4. Patent application numbers. S5 Distribution of papers per publishing institutions. S6. Applicants with active patent filing. S7. Top journals and individuals. [file 40104_2023_898_MOESM1_ESM.docx]

# Supplementary materials

## S1 Search methods and keys

### **Search key construction**

The technological landscape of papers and patents was respectively made using a scientific literature database (i.e., Web of Science) and a patent database (i.e., PATSTAT 2021b version), followed by an updated patent search, manually made using the Espacenet database. The data were extracted in May 2023. A composed search key was constructed starting from the relevant search terms combined with Boolean operators. Gradually, terms were added one by one and if an additional search term added relevant records to the dataset, it was retained in the key. This iterative process was repeated until the search key captured no new relevant literature without adding false positives. First, the patent search key was completed which was subsequently used to generate the paper search key. In the end, the two keys were not identical because of the different terminology used in each of the domains.

The search output was used to generate a patent and a paper dataset respectively and manual operations were performed to standardize the information. The patent citations were manually retrieved from the Web of Science and the legal statuses from Espacenet, the local patent offices, or PatSnap analytics (PatSnap analytics, London, UK). The patent search results were analyzed regarding their publication date and -number, the filed country, the patent family, the inventors, and the applicants. Next, the legal statuses were characterized as active (i.e., for granted patents with active status), pending (i.e., patents under examination before publication), rejected patents by the examiner, discontinued (i.e., patents abandoned before grant), ceased (i.e., patents lapsed or expired after being granted), and patents in which the fees were not paid. For the cases in which the legal status was not available, the countries’ local patent offices were consulted. For the scientific publications, the publication date, the institution, the country, the authors, the journal, and the citation numbers were considered for the analysis.

PATSTAT search key:

((upper(appln_title) like '%PRE_HATCH%' or upper(appln_title) like '%PREHATCH%' or upper(appln_title) like '%EMBRYO%EGG%' or upper(appln_title) like '%EGG%EMBRYO%' or upper(appln_title) like '%UNHATCH%EGG%' or upper(appln_title) like '%BIRD%EGG%' or upper(appln_title) like '%BIRD%EMBRYO%' or upper(appln_title) like '%AVIAN%EMBRYO%' or upper(appln_title) like '%AVIAN%EGG%' or upper(appln_title) like '%HEN% EGG%' or upper(appln_title) like '%HEN% EMBRYO%' or upper(appln_title) like '%CHICKEN% EGG%' or upper(appln_title) like '%CHICKEN% EMBRYO%' or upper(appln_title) like '%EMBRYONATED EGG%' or upper(appln_title) like '%POULTRY EMBRYO EGG%' or upper(appln_title) like '%EGG%BIRD%' or upper(appln_title) like '%EMBRYO%BIRD%' or upper(appln_title) like '%EGG %HEN%' or upper(appln_title) like '%EMBRYO %HEN%' or upper(appln_title) like '%EGG %CHICKEN%' or upper(appln_title) like '%EMBRYO %CHICKEN%')

and (upper(appln_title) like '%SEX%' OR upper(appln_title) like '%GENDER%'))

or upper(t.appln_title) like '%OVO%' and upper(t.appln_title) like '%SEXING%'

or upper(appln_title) like '%SEX OF AN EGG%'

Web of Science search key:

TS=((("PRE HATCH*" OR "BIRD EGG*" OR "BIRD EMBRYO*" OR "AVIAN EMBRYO*" OR "AVIAN EGG*" OR "HEN EGG*" OR "HEN EMBRYO*" OR "CHICKEN EGG*" OR "CHICKEN EMBRYO*" OR "EMBRYONATED EGG*" OR "POULTRY EMBRYO EGG*" OR "EGG HEN*" OR "EMBRYO HEN*" OR "EGG CHICKEN*" OR "EMBRYO CHICKEN*" OR "HATCHING EGG*" OR "BRED EGG*" OR ((EGG NEAR ODO*R) AND AVIAN)) AND ("SEX*" OR "GENDER*")) OR ("OVO" AND "SEXING") OR "SEX OF AN EGG" OR "sex in egg*" OR ((KILLING OR CULLING OR EUTHANASIA) NEAR (("DAY-OLD" OR Male) NEAR CHICK*)) OR ("SEX REVERSAL" AND CHICKEN))

## S2 Papers and patents related to immunosensing

**Table 1** Overview of papers and patents related to immunosensing strategies for in ovo sexing with hormones

| **Hormone** | **Sample** | **Detection (days)** | **Remarks** | **Reference** |
| --- | --- | --- | --- | --- |
| **Papers** | | | | |
| Luteinizing hormone, T, E2, P4 | Blood | 17 and 20 | Detection with RIA  Progesterone and estradiol with the bigger differences between males and females | **Tanabe et al.** [1] |
| E1, E2-17β, E2-17α sulphate, E1, E2-17β, E2-17α glucuronide | AF | 8 to 17 | Detection with RIA | **Gill et al.** [2] |
| T, A4 | Yolk |  | Differences were not sufficient for sex determination | **Müller et al.** [3] |
| E2 | AF | 17 | Detection with RIA  Significant differences | **Phelps et al.** [4] |
| E2-17ss | AF | 17 | Use of a yeast transactivation assay  Significant differences | **Tran et al.** [5] |
| T, E2, A4, P4, DHT | Yolk |  | Differences were not sufficient for sex determination | **Aslam et al.** [6] |
| E1S, E2, T | AF | 9 and 10 | Detection with ELISA  Significant differences for E1S on d 9 and for E2 on d 10 | **Weissmann et al.** [7] |
| E2, T, E1, A4, DHT | Serum | 8 to 16 | Detection with ELISA  Significant differences with T, DHT, and E1 | **Wang et al.** [8] |
| **Patents** | | | | |
| E2, E2-17ss, estriol, E1, T, DHT | AF | 11 to 19 |  | **Phelps** [9] |
| E2, E2-17ss, estriol, E1, T, DHT | AF | 11 to 19 |  | **Tyczkowski et al.** [10] |
| E2 | AF | 17 |  | **Butt and Tran** [11] |
| E1S | AF | 9 and 10 |  | **Einspanier** [12] |

A4 androstenedione, DHT dihydrotestosterone, E1 estrone, E2 estradiol, E1S estrone sulfate, P4 progesterone, T testosterone, RIA radioimmunoassay, ELISA enzyme-linked immunosorbent assay, AF allantoic fluid, d day.

## S3 Countries allowing or banning GMO importation

**Table S3** Overview of countries where import of GMOs is allowed or where a complete ban exists. The remaining group that allows both producing and importuning (or is without legislation regarding GMOs) is not mentioned in this table. Remarkably, within the EU, countries such as Spain and Portugal produce GMO maize [13]

| Importation is allowed | Complete ban |
| --- | --- |
| Azerbaijan, Belize, Bosnia and Herzegovina, Cyprus, Ecuador, Moldova, Norway, Saudi Arabia, Serbia, Switzerland, Turkey, France, Germany, Austria, Greece, Hungary, The Netherlands, Latvia, Lithuania, Luxemburg, Bulgaria, Poland, Denmark, Malta, Slovenia, Italy, Croatia, Wallonia, Scotland, Wales, Northern Ireland | Algeria, Bhutan, Kenya, Kyrgyzstan, Madagascar, Peru, Russia, Venezuela, Zimbabwe |

## S4 Patent application numbers


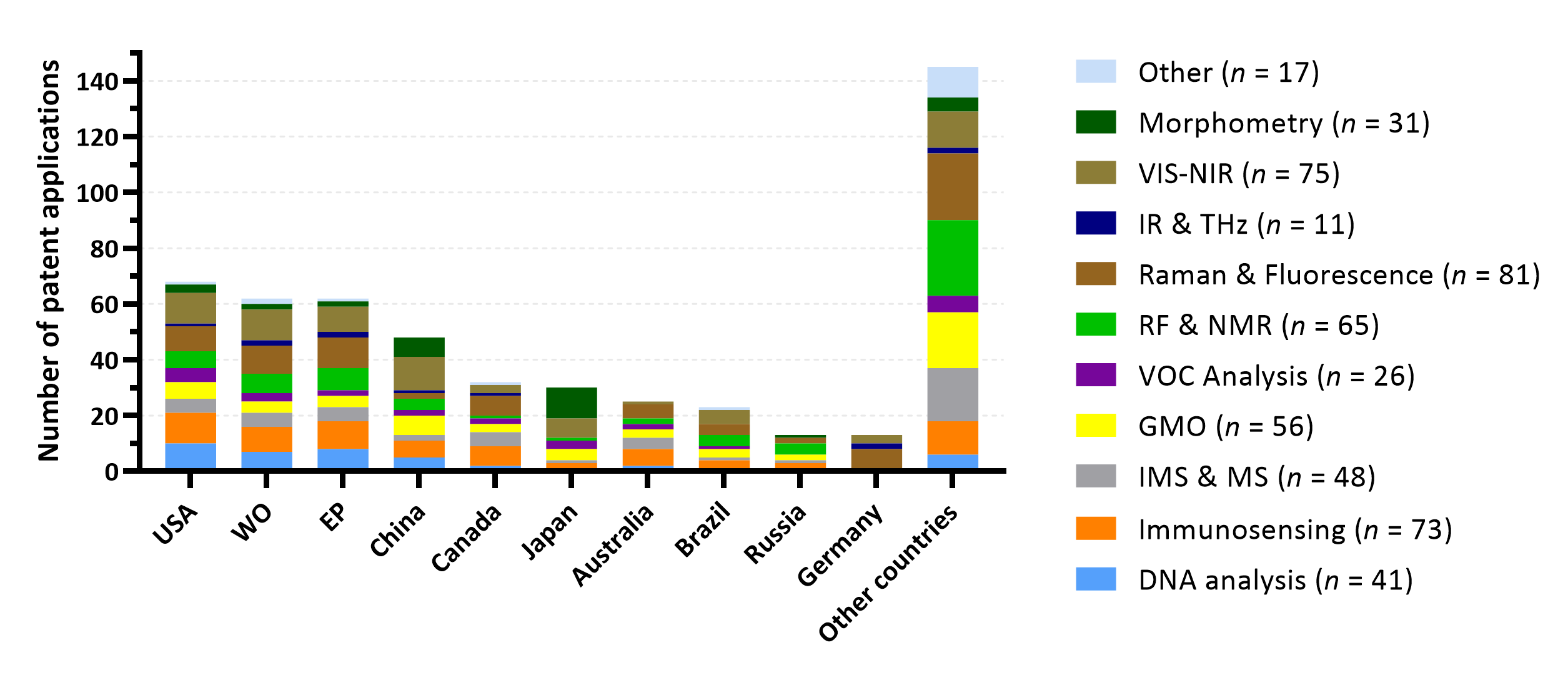


**Fig. S4** Country distribution of the published patents on in ovo sexing by their category. The figure depicts countries with 10 or more filed patents and also includes world (WO) and European patents (EP). A total of 524 patent applications on in ovo sexing was found. The categories were heterogeneously spread over regions such as the USA, WO, EP, China, and Canada. Japan predominantly protected morphometric and VIS-NIR approaches, whereas Germany focused on optical techniques only.

## S5 Distribution of papers per publishing institutions


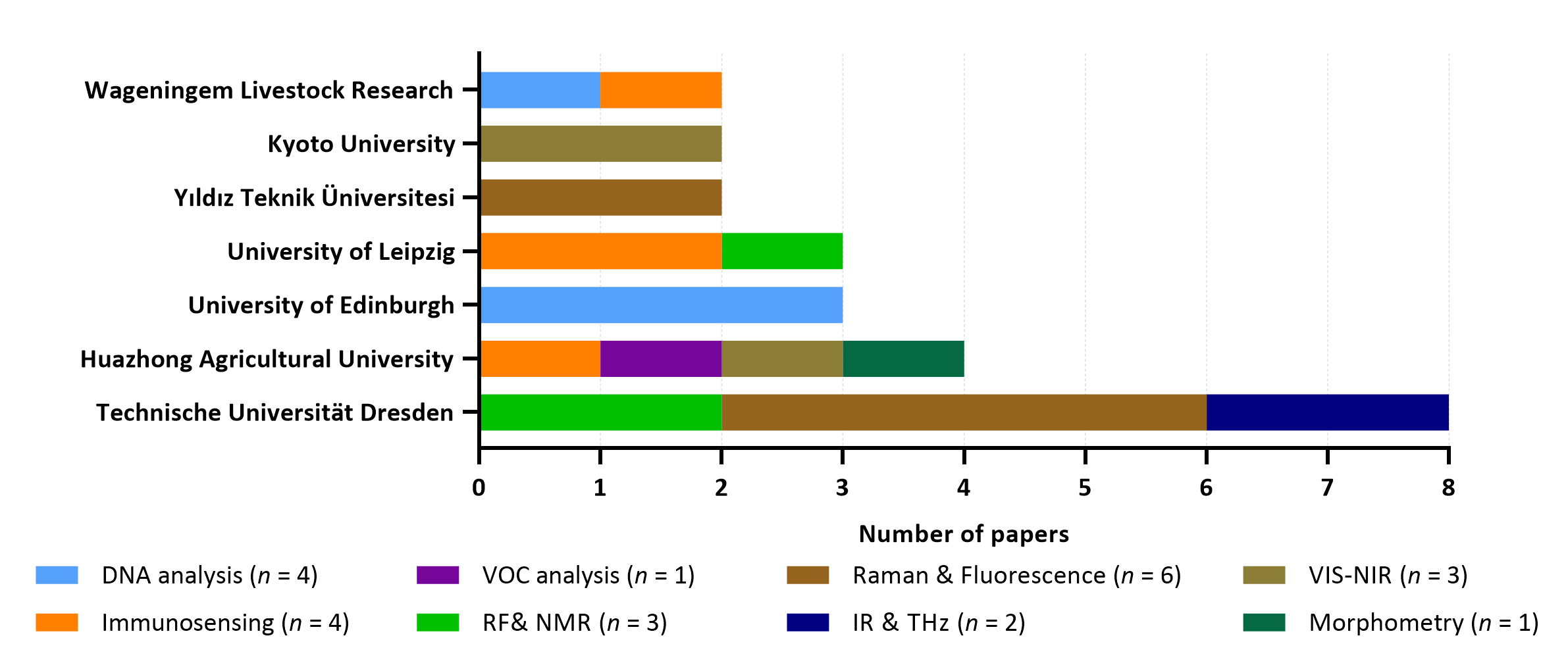


**Fig. S5** Distribution of the papers on in ovo sexing per institutions of origin and category. The total number of publications per category is indicated in between parentheses next to the category.

## S6 Applicants with active patent filing


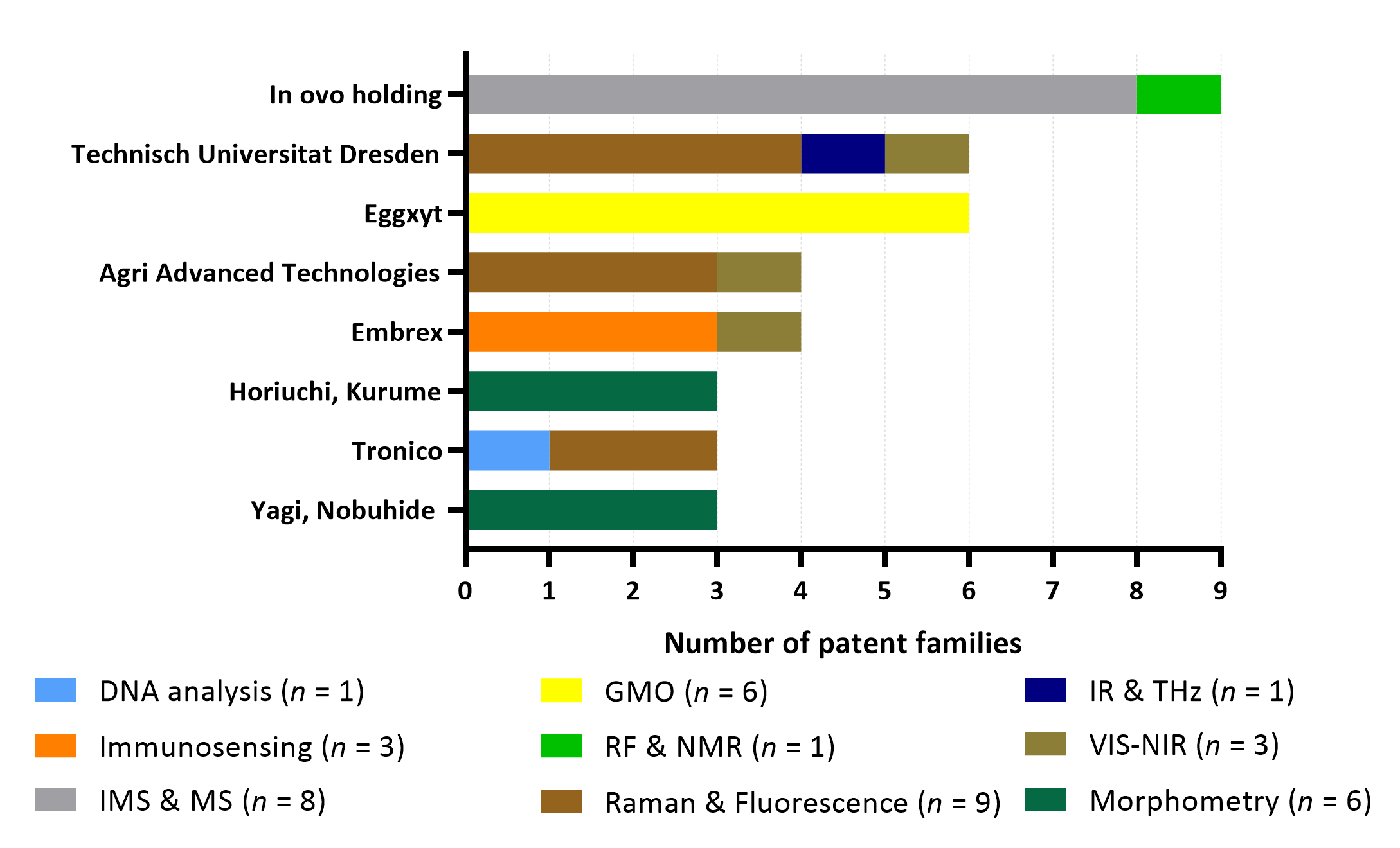


**Fig. S6** Distribution of the patent families regarding their applicants and category. The total number of patent applications per category is indicated in between parentheses next to the category.

## S7 Top journals and individuals


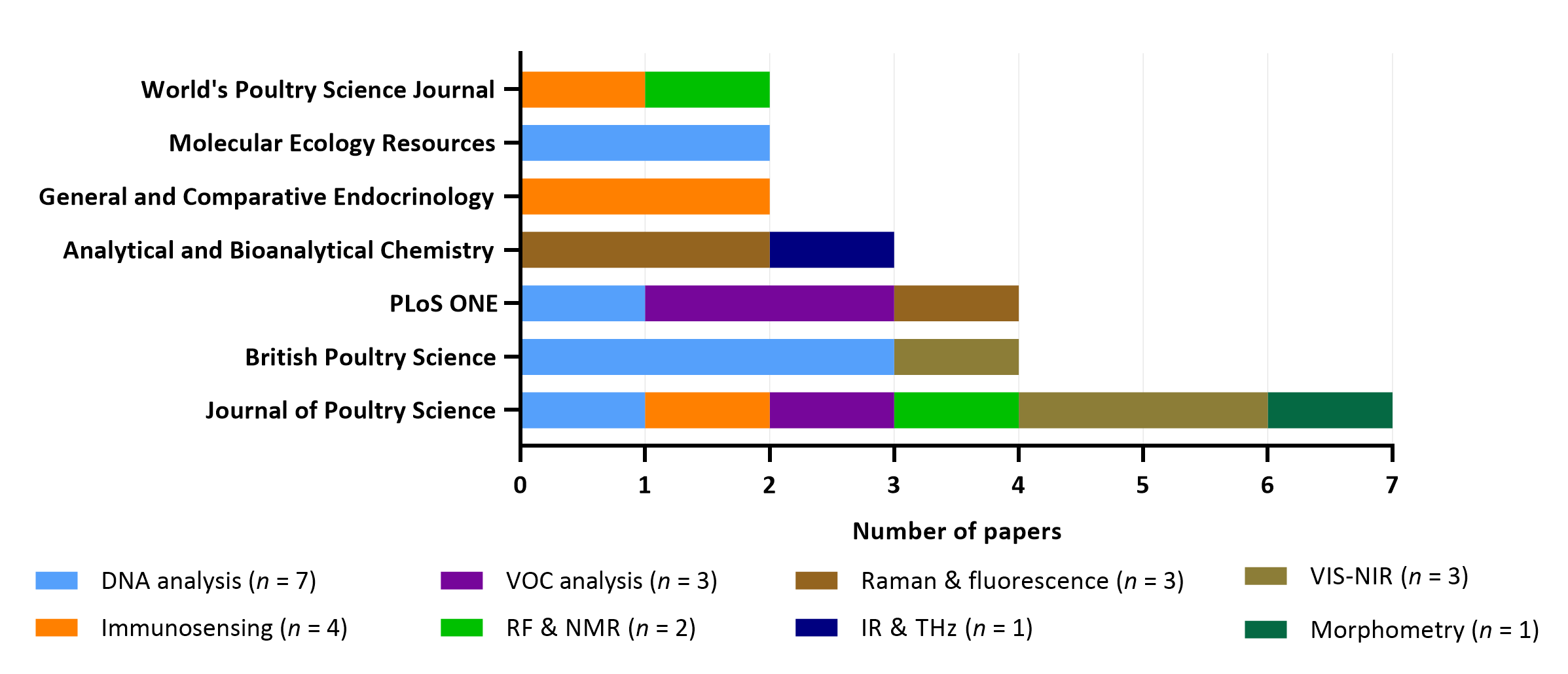


**Fig. S7** Paper distribution by the journal per category. Most of the journals were directly related to poultry (World's Poultry Science Journal, *n* = 2, British Poultry Science, *n* = 4, and Journal of Poultry Science, *n* = 7). Specific techniques could also be found in their related journals, such as DNA analysis papers published in Molecular Ecology Resources or immunosensing-related techniques published in General and Comparative Endocrinology.

## References

1. Tanabe Y, Nakamura T, Fujioka K, Doi O. Production and secretion of sex steroid hormones by the testes, the ovary, and the adrenal glands of embryonic and young chickens (Gallus domesticus). Gen Comp Endocrinol. 1979;39(1):26–33. https://doi.org/10.1016/0016-6480(79)90189-8.

2. Gill D V., Robertson HA, Betz TW. In vivo estrogen synthesis by the developing chicken (Gallus gallus) embryo. Gen Comp Endocrinol. 1983;49(2):176–86. https://doi.org/10.1016/0016-6480(83)90134-X.

3. Müller W, Eising CM, Dijkstra C, Groothuis TGG. Sex differences in yolk hormones depend on maternal social status in leghorn chickens (Gallus gallus domesticus). Proc R Soc B Biol Sci. 2002;269(1506):2249–55. https://doi.org/10.1098/rspb.2002.2159.

4. Phelps P, Bhutada A, Bryan S, Chalker A, Ferrell B, Neuman S, et al. Automated identification of male layer chicks prior to hatch. Worlds Poult Sci J. 2003;59(1):33–8.

5. Tran HT, Ferrell W, Butt TR. An estrogen sensor for poultry sex sorting. J Anim Sci. 2010;88(4):1358–64. https://doi.org/10.2527/jas.2009-2212.

6. Aslam MA, Hulst M, Hoving-Bolink RAH, Smits MA, de Vries B, Weites I, et al. Yolk concentrations of hormones and glucose and egg weight and egg dimensions in unincubated chicken eggs, in relation to egg sex and hen body weight. Gen Comp Endocrinol. 2013;187:15–22. https://doi.org/10.1016/j.ygcen.2013.02.045.

7. Weissmann A, Reitemeier S, Hahn A, Gottschalk J, Einspanier A. Sexing domestic chicken before hatch: A new method for in ovo gender identification. Theriogenology. 2013;80(3):199–205. https://doi.org/10.1016/j.theriogenology.2013.04.014.

8. Wang Y, Jin G, Ma M, Xiang X. Sex differences in serum steroid hormone levels during embryonic development in hen eggs. Poult Sci. 2019;98(11):6053–62. https://doi.org/10.3382/ps/pez270.

9. Phelps P. Method of sorting birds in ovo. WO9814781, 1998.

10. Tyczkowski J, Mahato D, Chalker A. Competitive particle immunoassay methods utilizing fluorescence microscopy. CA2607284A1, 2006.

11. Butt T, Tran HT. Compositions and methods for gender sorting. WO02086446A2, 2002.

12. Einspanier A. Method for the in-ovo sex identification of chicks. WO2017109133A1, 2017.

13. Güneş E, Movassaghi H, Unsal F, Güneş NT. GMO Policies and Practices: A Global Overview with Special Focus on Turkey. In: Policy Issues in Genetically Modified Crops. 2021. https://doi.org/10.1016/B978-0-12-820780-2.00002-9
